# A novel highly potent trivalent TGF-β receptor trap inhibits early-stage tumorigenesis and tumor cell invasion in murine Pten-deficient prostate glands

**DOI:** 10.18632/oncotarget.13343

**Published:** 2016-11-14

**Authors:** Tai Qin, Lindsey Barron, Lu Xia, Haojie Huang, Maria M. Villarreal, John Zwaagstra, Cathy Collins, Junhua Yang, Christian Zwieb, Ravindra Kodali, Cynthia S. Hinck, Sun Kyung Kim, Robert L. Reddick, Chang Shu, Maureen D. O’Connor-McCourt, Andrew P. Hinck, Lu-Zhe Sun

**Affiliations:** ^1^ Department of Cell Systems and Anatomy, University of Texas Health Science Center, San Antonio, TX, USA; ^2^ Department of Vascular Surgery, Second Xiangya Hospital and Xiangya School of Medicine, Central South University, Hunan, China; ^3^ Department of Gynecology and Obstetrics, Xiangya Hospital and Xiangya School of Medicine, Central South University, Hunan, China; ^4^ Department of Biochemistry, University of Texas Health Science Center, San Antonio, TX, USA; ^5^ Department of Biochemistry and Molecular Biology, Mayo Clinic College of Medicine, Rochester, MN, USA; ^6^ Department of Pathology, University of Texas Health Science Center, San Antonio, TX, USA; ^7^ Cancer Therapy and Research Center, University of Texas Health Science Center at San Antonio, Texas, USA; ^8^ Department of Structural Biology, University of Pittsburgh School of Medicine, Pittsburgh, PA USA; ^9^ National Research Council Human Health Therapeutics Portfolio, Montréal, Quebec, Canada, Maureen O'Connor-McCourt is currently affiliated with Formation Biologics, Montréal, Quebec, Canada

**Keywords:** TGF-β trap, RER, tumorigenesis, Pten, prostate cancer

## Abstract

The effects of transforming growth factor beta (TGF-β) signaling on prostate tumorigenesis has been shown to be strongly dependent on the stage of development, with TGF-β functioning as a tumor suppressor in early stages of disease and as a promoter in later stages. To study in further detail the paradoxical tumor-suppressive and tumor-promoting roles of the TGF-β pathway, we investigated the effect of systemic treatment with a TGF-β inhibitor on early stages of prostate tumorigenesis. To ensure effective inhibition, we developed and employed a novel trivalent TGF-β receptor trap, RER, comprised of domains derived from the TGF-β type II and type III receptors. This trap was shown to completely block TβRII binding, to antagonize TGF-β1 and TGF-β3 signaling in cultured epithelial cells at low picomolar concentrations, and it showed equal or better anti-TGF-β activities than a pan TGF-β neutralizing antibody and a TGF-β receptor I kinase inhibitor in various prostate cancer cell lines. Systemic administration of RER inhibited prostate tumor cell proliferation as indicated by reduced Ki67 positive cells and invasion potential of tumor cells in high grade prostatic intraepithelial neoplasia (PIN) lesions in the prostate glands of Pten conditional null mice. These results provide evidence that TGF-β acts as a promoter rather than a suppressor in the relatively early stages of this spontaneous prostate tumorigenesis model. Thus, inhibition of TGF-β signaling in early stages of prostate cancer may be a novel therapeutic strategy to inhibit the progression as well as the metastatic potential in patients with prostate cancer.

## INTRODUCTION

Prostate cancer (PCa) is the leading cancer diagnosed and the second cause of cancer related death in American men. It has been estimated by the American Cancer Society that about 1 man in 7 will be diagnosed with prostate cancer during his lifetime and about 1 man in 38 will die of prostate cancer [1]. The mortality mainly results from progression of androgen-dependent to androgen-independent tumor growth after androgen deprivation therapy and metastasis to various organs that involves activation of multiple oncogenic pathways.

Transforming growth factor beta (TGF-β) isoforms, TGF-β1, β2, and β3, are small (25 kDa) homodimeric signaling proteins. They are secreted in a latent form and are activated by multiple mechanisms, including integrin binding and proteolysis [2]. They form a complex with the TGF-β type I and type II receptors (TβRI and TβRII) for signal transduction, in which TβRII phosphorylates and activates TβRI [3]. The phospho-TβRI then phosphorylates intracellular Smad2 and Smad3, which form a complex with the common-mediator Smad, Smad4, to regulate gene expression [4].

TGF-βs are potent growth inhibitors in normal epithelial cells, including normal prostate epithelial cells, by stimulating apoptosis and inhibiting G1 to S cell cycle progression [5]. The deletion of Smad4 has been furthermore shown to drive the invasion and metastasis of indolent prostate tumors with *Pten* deletion in a mouse model, demonstrating the tumor suppressive activity of the TGF-β/Smad pathway in the prostate gland [6]. Thus, it is not surprising that carcinoma cells in general and PCa cells in particular are resistant to TGF-β-induced growth inhibition and that loss of or reduced expression of the signaling receptors, TβRI, TβRII, or the non-signaling TGF-β type III receptor, also known as betaglycan, is often observed during the progression of human PCa [7–10].

Prostate carcinoma cells, while responding poorly to TGF-β-mediated growth inhibition, often produce much higher levels of TGF-β isoforms than their normal counterparts [11]. Furthermore, latent TGF-β is activated by the protease prostate specific antigen (PSA), which is an androgen receptor (AR) target gene abundantly secreted by advanced androgen-independent PCa cells [12]. Indeed, serum TGF-β1 levels have been shown to correlate with tumor burden, metastasis, and serum PSA in PCa patients and an increased level of TGF-β1 is strongly associated with PCa progression and poor clinical outcome [13, 14]. These observations suggest that excessive levels of TGF-β may act on tumor stromal cells in a paracrine fashion to promote disease progression.

TGF-β’s tumor promoting activity may be related to its ability to generate and maintain cancer stem cells, including PCa stem cells, which are AR negative and presumably sensitive to TGF-β [15]. TGF-βs are also known to stimulate the conversion of CD4^+^CD25^-^ T cells to CD4^+^CD25^+^Foxp3^+^ regulatory T-cells [16], which inhibit anti-tumor immunity. Treatments with TGF-β inhibitors, such as soluble betaglycan or a pan-isoform neutralizing antibody, have been shown to have beneficial effects in animal models of PCa, including inhibition of the growth and angiogenesis of tumors formed by AR negative human PCa cells [17] or inhibition of regulatory T-cell production and tumor progression [18]. Thus, there are multiple mechanisms by which TGF-βs promote the progression of advanced disease and treatment with TGF-β inhibitors appears to be a viable strategy for attenuating disease progression.

The TGF-β pathway is known however to be tumor suppressive in normal and some experimental models of early stage adenocarcinomas as mentioned above, and even advanced tumors may contain early and late stages of lesions due to tumor heterogeneity. Thus the greatest perceived risk of TGF-β antagonists in treating late stage PCa is the potential progression of early-stage tumor cells in which TGF-β pathway is still tumor suppressive. Here we investigate the consequences of TGF-β inhibition in a relatively early stage PCa model using a novel highly potent trivalent TGF-β receptor trap, known as RER. RER binds and antagonizes TGF-β at near picomolar concentrations and has advantages over kinase inhibitors and antibodies, including increased antagonistic potency and specificity. To fully assess the benefits, as well as any detrimental consequences of TGF-β inhibition, the effects of this inhibitor were evaluated in immune competent mice bearing a conditional deletion of *Pten* in the prostate epithelium. These animals develop prostatic intraepithelial neoplasia (PIN) lesions in a time-dependent manner that closely recapitulates human disease [19]. The results showed that systemic treatment with RER unexpectedly inhibited tumor cell proliferation in high grade PIN lesions in 6–8 month old mice, indicating that TGF-β in the high grade PIN microenvironment acts to promote neoplastic cell proliferation. Treatment with RER also inhibited stromal invasion by tumor cells. These results suggest that TGF-β’s tumor-promoting function may occur at a relatively early stage during prostate tumorigenesis and RER may serve as a potential TGF-β inhibitor for treating early stage disease.

## RESULTS

### Novel trivalent TGF-β receptor trap RER

**Figure 1:**
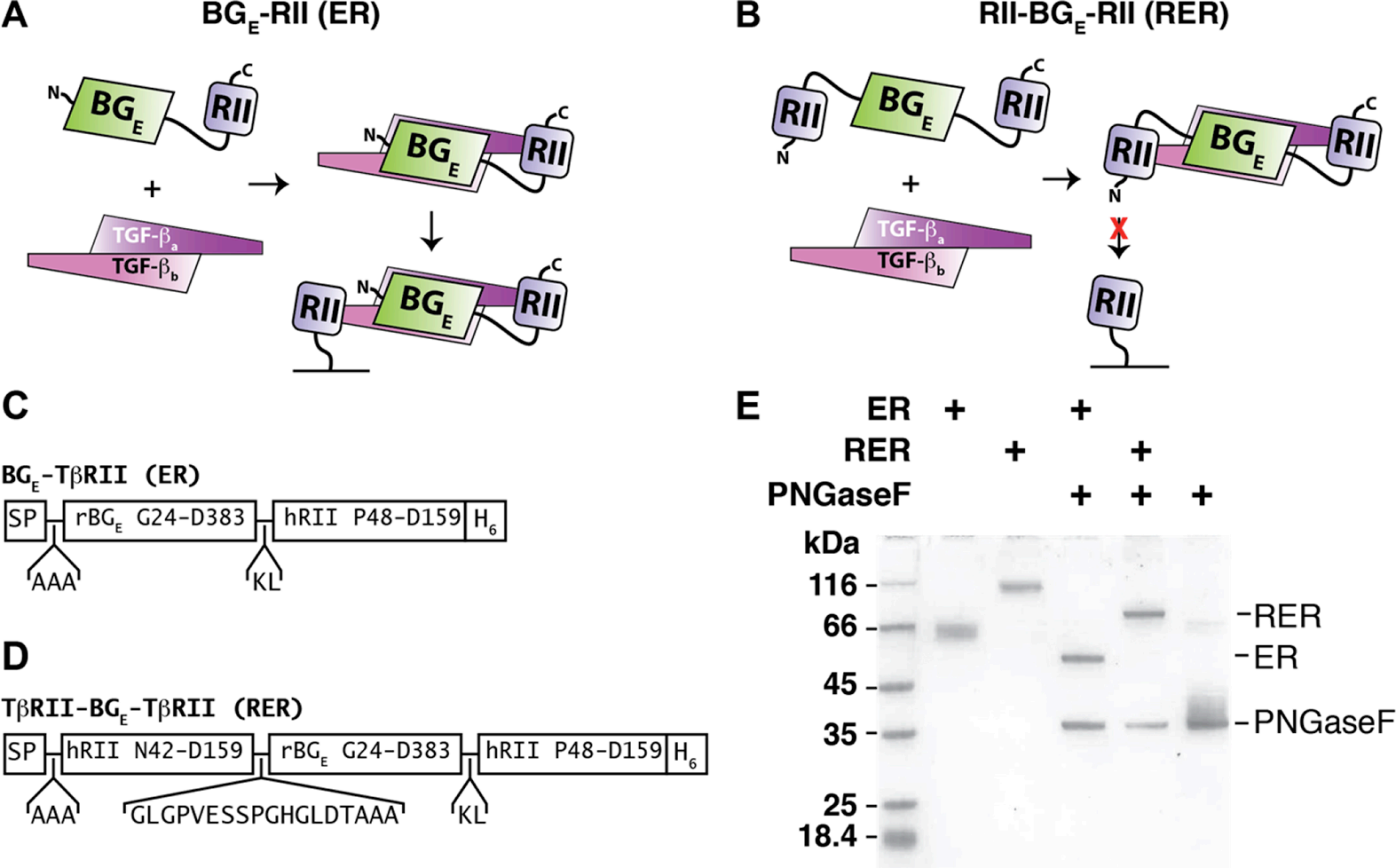
ER and RER expression and purification (**A**–**B**) Schematic diagram of the bivalent ER (A) and trivalent RER (B) receptor traps and proposed manner of binding to TGF-β homodimers. ER-bound TGF-β, yet not RER-bound TGF-β, is predicted to bind to membrane-bound TβRII. (**C**–**D**) Schematic diagram of the ER (C) and RER (D) expression constructs. SP and H_6_ correspond to the rat serum albumin signal peptide and histidine tag, which have the sequences MKWVTFLLLFISGSAFS and HHHHHH, respectively. (**E**) SDS-PAGE of purified as isolated (glycosylated) or PNGaseF-treated (deglycosylated) ER and RER. Samples were not reduced prior to loading onto the gel.

We previously reported an engineered bivalent TGF-β receptor trap protein known as BG_E_-RII and demonstrated that it had improved antagonistic potency against all three TGF-β isoforms compared to its two component binding domains, the N-terminal TGF-β binding endoglin-like domain of the TGF-β co-receptor betaglycan (BG_E_) and the TGF-β type II receptor extracellular domain (RII) [21]. We know from structures that TGF-βs bind RII symmetrically to form 1:2 complexes [21–23] and that BG_E_ weakly cooperates with RII for binding TGF-βs (M. Villarreal et al, under review). We therefore reasoned that it might be possible to improve the antagonistic potency of BG_E_-RII by tethering an additional RII binding domain onto its N-terminus. One way in which the potency of the trivalent inhibitor RII-BG_E_-RII, or RER, might be improved relative to the bivalent inhibitor BG_E_-RII, or ER, is by the additive effects of multivalent binding. Another is by blocking any ability of the bound TGF-β to bind cell surface RII (Figure 1A–1B).

We constructed the coding sequence for the bivalent trap ER and a homologous sequence for the trivalent trap RER and placed these downstream of the rat serum albumin signal peptide in a CMV-based mammalian expression vector (Figure 1C–1D, Supplementary Figures S1 and S2, and Supplementary Figures S2 and S3). We produced ER and RER, which have 8 and 14 disulfide bonds, respectively, in suspension-cultured HEK293 Freestyle (HEK293F) cells and were able to recover 50–100 mg quantities per liter after purification. Purified ER and RER had slower than expected mobilities (ER: 54 kDa expected, 63 kDa observed; RER 69 kDa expected, 98 kDa observed) when analyzed by SDS-PAGE under non-reducing conditions (Figure 1E), consistent with the presence of multiple sites for N-linked glycosylation (Supplementary Figures S2 and S3). We confirmed the presence of the glycans by incubating purified ER and RER with PNGase-F under partially denaturing conditions and showed that this converted the recombinant proteins to a single sharp band that migrated at the expected size for their protein core (Figure 1E).

**Figure 2:**
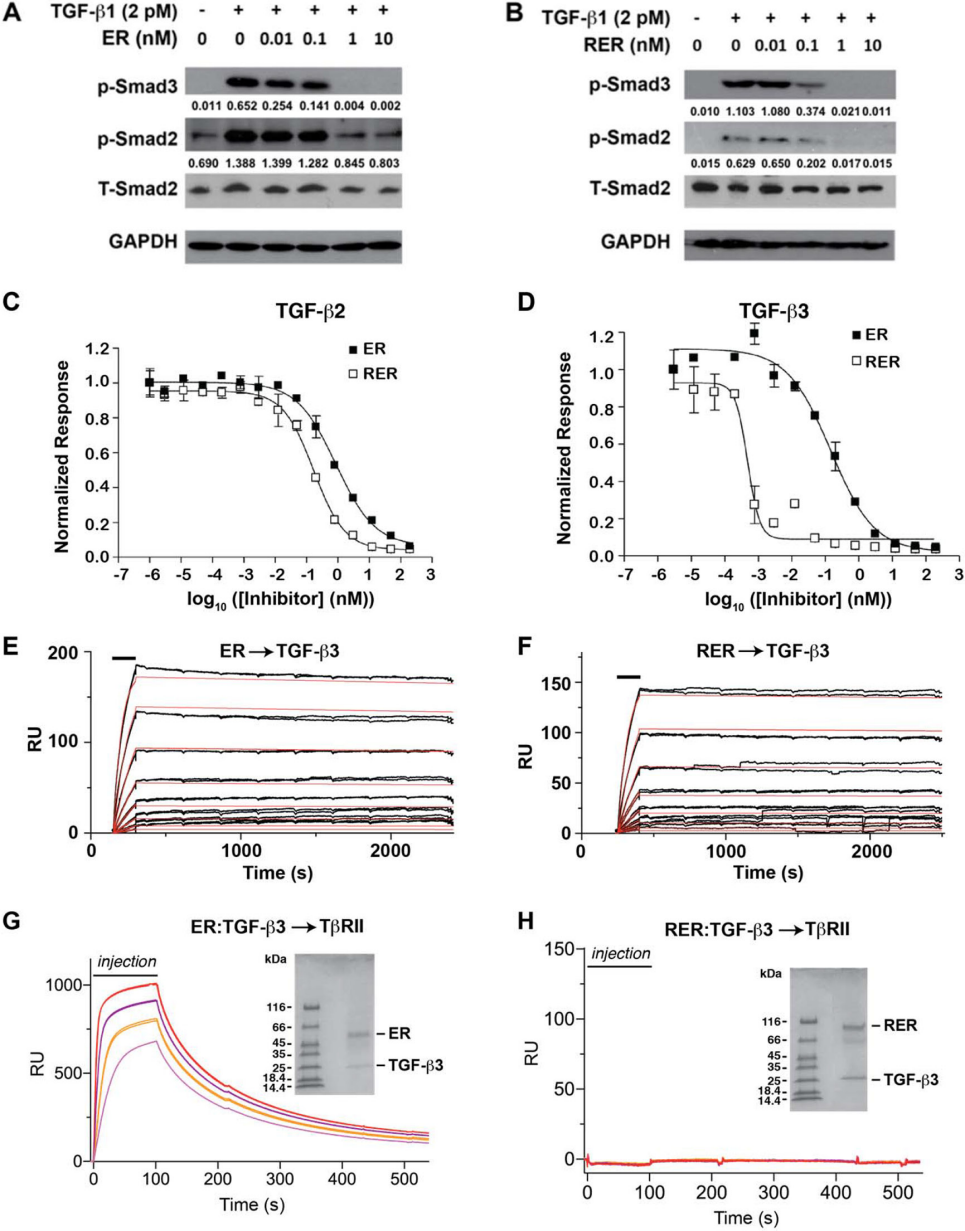
ER and RER antagonism of TGF-β signaling and mechanism of action (**A**–**B**) Western blots for phopho-Smad2 (p-Smad2) and phospho-Smad3 (p-Smad3) to qualitatively assess the potency of the ER and RER receptor traps as TGF-β1 antagonists. Blots were stripped and re-probed using either a Smad2 antibody (total Smad2 or T-Smad2) or glyceraldehyde-3-phosphate dehydrogenase (GAPDH) to control for equal loading. The value under each p-Smad2 and p-Smad3 band was normalized-density by the density of the corresponding T-Smad2 band with ImageJ program. (**C**–**D**) TGF-β PAI-1 luciferase reporter activity upon treatment of stably transfected cultured mink lung epithelial cells with 20 pM TGF-β2 or TGF-β3 as a function of increasing receptor trap concentration. Data points and associated error bars correspond to the mean and standard deviation among triplicate measurements. Smooth lines correspond to fits of the data to a standard equation for inhibition with variable slope. (**E**–**F**) Injection of the ER or RER receptor trap as a two-fold dilution series (12.5–400 pM) over immobilized TGF-β3. Injections were performed in triplicate and are indicated by black bars. Raw sensorgrams are shown in black. Global fit of the raw data to a 1:1 binding model is shown as smooth red curves. (**G**–**H**) SPR sensorgrams for injection of 0.5 μM (pink), 1 μM (orange), 2 μM (purple), and 4 μM (red) pre-purified TGF-β3:ER or TGF-β3:RER complexes over immobilized TβRII (G and H, respectively). SDS-PAGE gels of the injected complexes are shown in the insets.

### ER and RER inhibition of TGF-β signaling

Smad2 and Smad3 phosphorylation was measured after stimulation with 2 pM TGF-β1 in the presence of 0, 0.01, 0.1, 1 and 10 nM ER or RER to qualitatively compare the effects of the inhibitors on TGF-β signaling. ER was shown to reduce Smad2 phosphorylation by roughly 30% between concentrations of 0.1–1 nM (Figure 2A), while RER reduced Smad2 phosphorylation by about 70% between concentrations of 0.01–0.1 nM. p-Smad3 was similarly reduced more by RER than by ER at 0.1 nM(Figure 2B). Quantitative assessments of the antagonistic potency of ER and RER were obtained by measuring luciferase activity after induction of a stably transfected plasminogen activator inhibitor-1 (PAI-1) luciferase reporter in cultured Mv1Lu mink lung epithelial cells with 20 pM TGF -β1,-β2,-β3 in the presence of serial 4-fold dilutions of ER or RER. Measured luciferase values were normalized relative to a TGF-β only treated control and then fit to a standard binding equation with variable slope to obtain IC_50_ values (Figure 2C–2D). Measured IC_50_ values, which are the result of averaging the results from either 2 or 3 experiments, are listed in Supplementary Table S2. Consistent with the results from the Smad phosphorylation assay, RER was shown to be a more potent TGF-β1 antagonist than ER, with an approximate 30-fold lower IC_50_ value (0.5 ± 0.2 pM vs. 14 ± 9 pM). RER was also a more potent TGF-β2 or TGF-β3 antagonist than ER, although in these cases the differences were not as pronounced (70 ± 18 pM vs. 1200 ± 300 pM for TGF-β2 and 3.3 ± 5.8 vs. 20 ± 11 pM for TGF-β3).

### ER and RER mechanism of action

Kinetic surface plasmon resonance (SPR) experiments were performed to assess the kinetics and affinity with which ER and RER bind TGF-βs (Supplementary Figures S4 and S5). As shown in Figure 2E–2F, ER and RER both bound TGF-β3 rapidly, but disassociated very slowly, consistent with attenuation of the dissociation due to multivalent binding. Although the differences were subtle, RER was shown to disassociate roughly two-fold more slowly than ER, which translated into a roughly two-fold greater affinity as the association rates were similar (Figure 2E–2F and Supplementary Table S3). Although ER and RER both bound TGF-β2 about 30-fold more weakly than TGF-β3, the same overall pattern was observed, with RER disassociating about two-fold more slowly and the overall affinity being about two-fold greater (Supplementary Figure S4E, S4G, Supplementary Table S3). ER’s antagonistic potency in the luciferase assay is comparable to its K_D_ for binding (IC_50_s of 1200 and 20 pM and K_D_s of 1440 and 51 pM, respectively, for TGF-β2 and TGF-β3), while RER’s is not (IC50s of 70 and 3.3 pM and K_D_s of 823 and 24 pM, respectively for TGF-β2 and TGF-β3), suggesting that other factors, such as its ability to fully block TβRII binding might contribute its potency. To investigate the ability of ER and RER to block TβRII binding, ER:TGF-β3 or RER:TGF-β3 complexes were pre-formed by adding 1.1 molar equivalents of ER or RER to 1 equivlant of TGF-β3 followed by isolation of the complexes using size exclusion chromatography. Preformed complexes were then injected over immobilized TβRII (Supplementary Figure S6). Injection of the preformed ER:TGF-β3 complex resulted in a robust concentration-dependent response, while injection of the RER:TGF-β3 complex did not (Figure 2G, 2H). This suggests that the increased potency of RER over ER is derived both from increased affinity due to multivalent binding and ability to block TβRII binding, although in the context of cultured cells, the latter appears to be more important than the former.

### TGF-β inhibitors had varying efficacy in blocking TGF-β-induced Smad phosphorylation, cell growth inhibition, and migration

**Figure 3:**
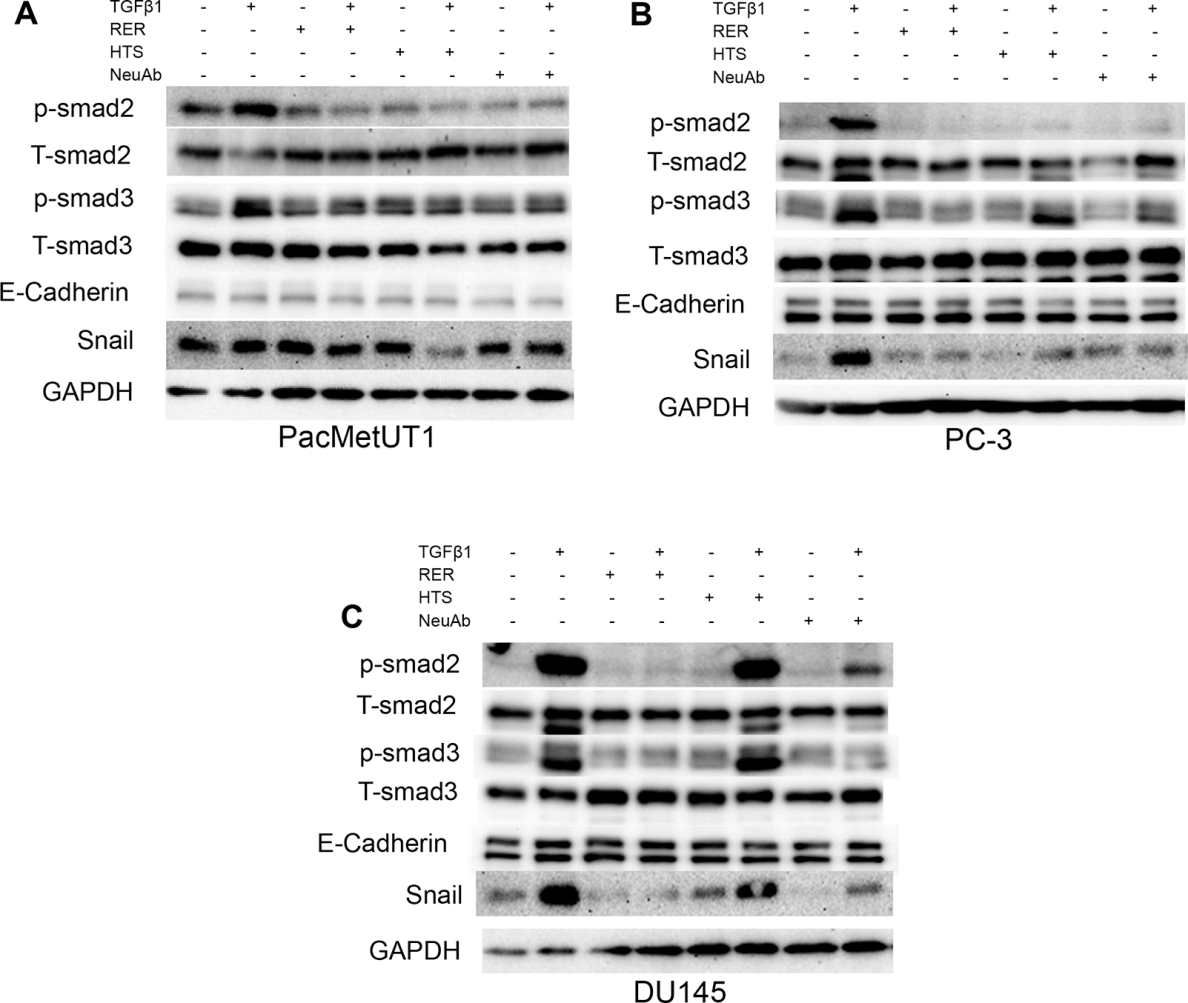
TGF-β inhibitors had varying efficacy in blocking TGF-β-induced Smad phosphorylation Western blotting analysis of phosphorylated Smad2 (p-Smad2), phosphorylated Smad3 (p-Smad3), total Smad2/3 (T-Smad2/3), E-cadherin, Snail expression in the PacMetUT1 (**A**), PC-3 (**B**) and DU145 (**C**) prostate cancer cell lines individually or in combination treated with drugs as indicated (TGF-β1 at 80 pM, RER at 50 nM, HTS at 50 nM, and TGF-β neutralizing antibody (NeuAb) at 50 nM) for 2 hrs. Glyceraldehyde-3- phosphate dehydrogenase (GAPDH) expression level was used to validate equal sample loading.

**Figure 4:**
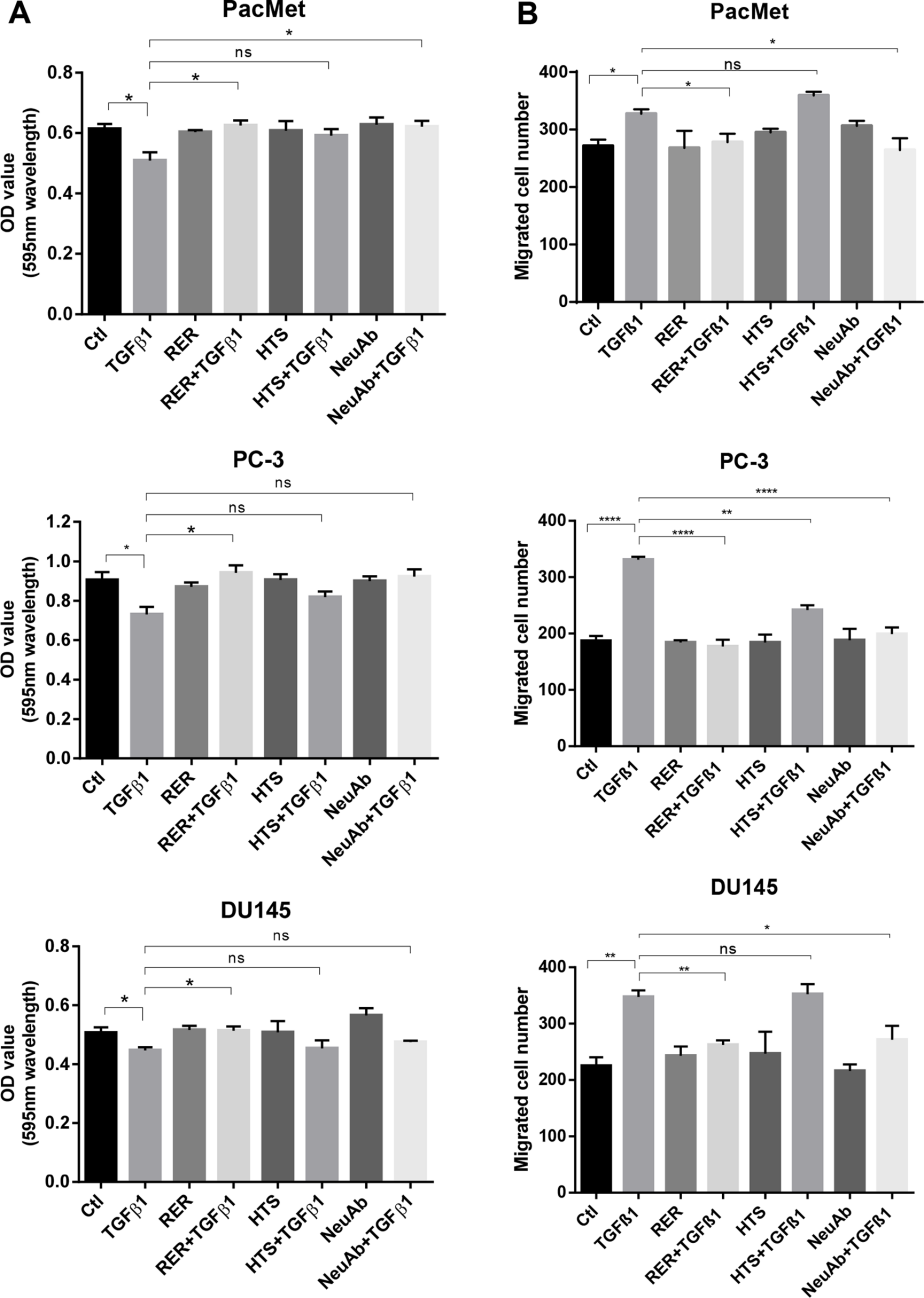
TGF-β inhibitors had varying efficacy in blocking TGF-β-induced cell growth inhibition and migration (**A**) PacMetUT1, PC-3 and DU145 cells were seeded in 96-well plates at 2,000 cells/well for 5hrs and treated with TGF-β1 (5 ng/ml), RER (100 nM), HTS (100 nM) and NeuAb (100 nM) for 5 days. MTT assay was performed to obtain OD values reflecting relative cell number. Each data bar represents the mean±SEM from three wells. (**B**) PacMetUT1, PC-3 and DU145 cells were plated in 24-well cell migration inserts at 40,000cells/insert treated with TGF-β1 (2 ng/ml), RER (50 nM), HTS (50 nM) and NeuAb (50 nM) alone or in combination in medium. Migration assay was performed after 16 h for PacMetUT1 and PC3 cells and 8h for DU145 cells. Migrated cells in each insert were counted under microscope. Data presented are mean±SEM from triplicate wells. *P <0.05, **P <0.01, ***P < 0.0001.

As widely reported, TGF-β can inhibit the cell proliferation, but promote cell migration in various cell types [24, 25]. We next investigated if RER as a potent TGF-β ligand trap could attenuate these activities of TGF-β in prostate cancer cells and compared its potency with other types of TGF-β inhibitors that have been used in preclinical models including a TβRI kinase inhibitor (HTS) and the 1D11 pan TGF-β neutralizing antibody (NeuAb). We treated the human prostate cancer cell lines PacMetUT1, PC-3, and DU145 with TGF-β1, RER, HTS, or NeuAb alone, or TGF-β1 in combination with RER, HTS, or NeuAb for 2 hrs. We found that while the three TGF-β inhibitors were all effective in blocking TGF-β-induced phosphorylation of Smad2 and Smad3, RER appeared to be most effective in the PC-3 and DU145 cells, which are more sensitive to TGF-β1 than PacMetUT1 cells (Figure 3A–3C). Consistently, RER was equally or most effective in blocking TGF-β1-induced expression of Snail, a TGF-β target gene and an epithelial to mesenchymal transition (EMT) marker [26] (Figure 3A–3C). Interestingly, the expression of another EMT marker E-cadherin was not affected by either TGF-β1 or TGF-β inhibitor treatment, most likely because of the short-term treatment as it was reported by Maeda and coworkers [27] that the down-regulation of E-cadherin expression happened after TGF-β1 treatment for 4 days in cancer cells. We then investigated if the inhibition of TGF-β signaling by the three TGF-β inhibitors could impact cell behavior. We performed the proliferation and migration assays in the three cell lines, and found that RER was again most effective in blocking the moderate inhibition of cell proliferation by TGF-β in the three cell lines (Figure 4A). For cell migration, NeuAb appeared equally effective as RER in blocking TGF-β1-induced migration in all cell lines (Figure 4B). HTS appeared least active in these assays. Taken together, these data show that RER is an effective TGF-β inhibitor *in vitro*.

### RER treatment attenuated TGF-β signaling *in vivo*

**Figure 5:**
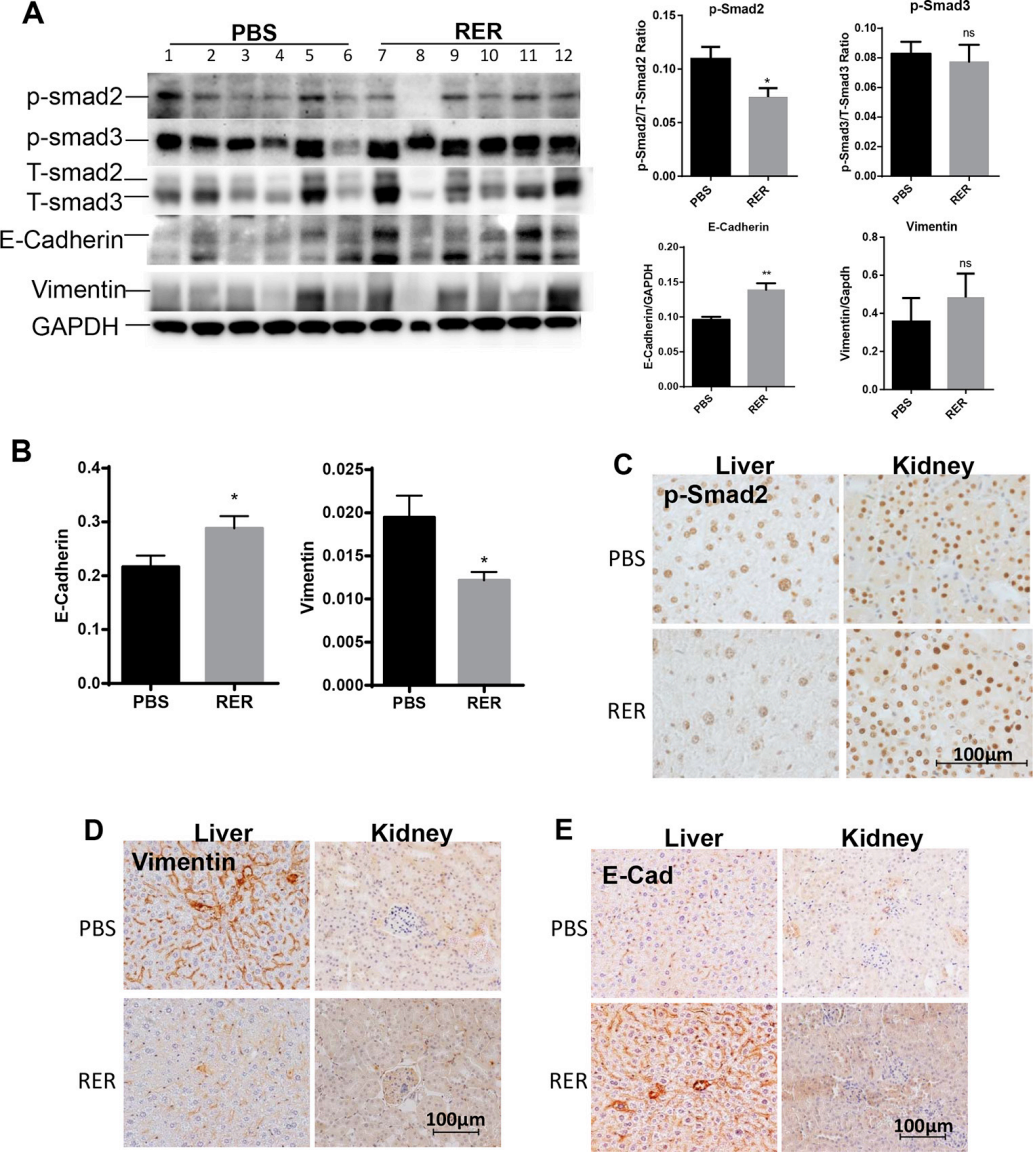
RER treatment attenuated TGF-β signaling in vivo (**A**) Western blot analysis of p-Smad2, p-Smad3, T-Smad2/3, E-cadherin, Vimentin in anterior prostate (AP) from all the experimental mice as indicated. The bar plots are the total Smad2 or Smad3-normalized p-Smad2 or p-Smad3 levels and the GAPDH-normalized E-cadherin and Vimentin for each sample quantified with Protein Simple software. Each data bar represents the mean±SEM from six samples. (**B**) qRT-PCR analysis of mRNA expression of E-cadherin and Vimentin in dorsal and ventral prostate of all the experimental mice. (**C**, **D**, **E**) IHC staining of p-Smad2, Vimentin, and E-cadherin in liver and kidney tissue of the experimental mice. A representative picture was randomly taken for each staining from tissue sections of three mice in each group.

We next applied RER *in vivo* to explore if it could inhibit TGF-β signaling and impact prostate tumorigenesis and tumor progression. We used the 6–8 month-old prostate specific *Pten* knockout mice (Pten KO) to investigate the role of TGF-β signaling in relatively early/intermediate stage of prostate tumorigenesis as Pten KO mice in this age group develop high grade prostatic intraepithelial (PIN) lesions and focal invasive adenocarcinoma (Supplementary Figure S6) [19]. Intraperitoneally injected RER at 50 μg/mouse/day for 30 days was detectable in serum, prostate, liver and kidney as shown in Supplementary Figure S7 and described in Supplementary Figure S7. To investigate whether the treatment with RER abrogated TGF-β signaling *in vivo*, we conducted immunoblotting, qRT-PCR, and IHC to gain molecular insights into biological processes impacted by RER. Immunoblotting for Smad2, Smad3, E-cadherin, and vimentin in the anterior lobes of the prostate glands showed a pattern of reduced P-Smad2 levels and increased E-cadherin levels in the RER group in comparison with the control group (Figure 5A, left). Quantification of the density of the bands and normalization P-Smad and E-cadherin to the corresponding T-Smad and GAPDH, respectively, revealed that the decrease of P-Smad2 and the increase of E-Cadherin in the RER group were statistically significant (Figure 5A, right). On the other hand, the levels of P-Smad3 and vimentin, which can be increased by TGF-β, were not altered after RER treatment suggesting that they might be less sensitive to the treatment than P-Smad2 and E-cadherin. Using RT-PCR, we also found that E-cadherin mRNA levels in DP plus VP were significantly increased and vimentin mRNA levels were significantly decreased in the RER group (Figure 5B). Since RER was readily detectable in both liver and kidney, we also examined whether RER was active in these two tissues in inhibiting TGF-β signaling. By performing IHC, we found decreased levels of p-Smad2 (Figure 5C) and vimentin (Figure 5D), and increased E-cadherin (Figure 5E) in the liver tissues after RER treatment. In contrast, these changes were not as obvious in the kidney tissues (Figure 5C–5E) suggesting RER might not be as active in the kidney. The treatment with RER also did not change body weight. Thus, RER treatment was effective in inhibiting TGF-β activity in certain tissues *in vivo* without noticeable toxicity.

### RER treatment moderately blocked early stage prostate cancer progression *in vivo*

**Figure 6:**
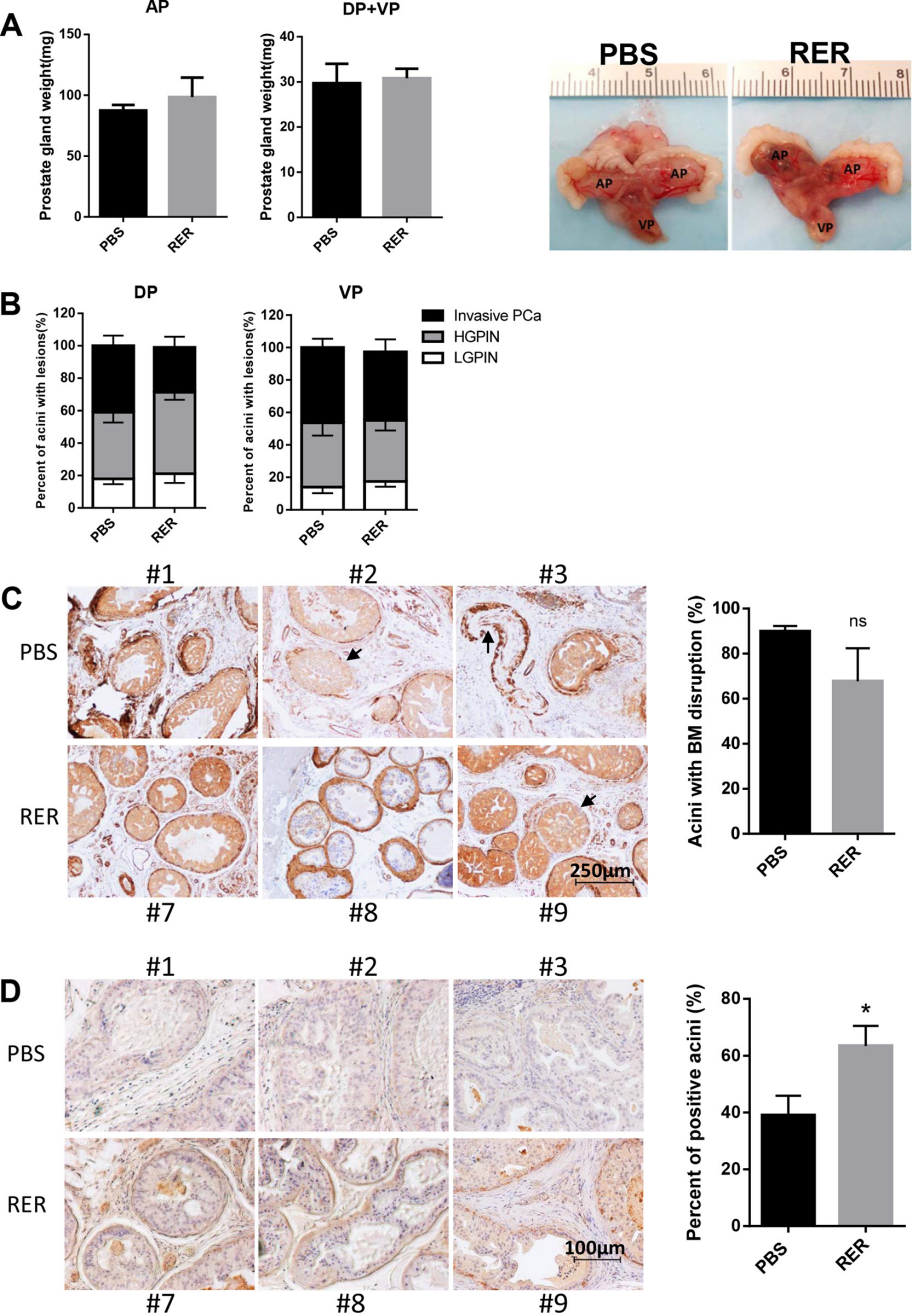
RER treatment moderately blocked early stage prostate cancer progression in vivo (**A**) Each prostate gland was isolated from mice and weight was recorded. Bar plots show the weight of prostate glands in PBS and RER group. Representative pictures of prostate glands from PBS and RER-treated mice are presented. (**B**) Percent of normal/low grade PIN lesion, high grade PIN lesion, and invasive adenocarcinoma in each prostate lobe was obtained by dividing the number of acini with the specific lesion by the total number of acini observed in one tissue section. (**C**) IHC analyses of α-SMA in DP lobes showed that RER treatment group had a moderately less breakdown of a continuous layer of basement membrane (BM) around each acinus. (**D**) IHC analysis of laminin in DP lobes showed that more acini in RER group had positive laminin staining than in Control group. All plotted data represent mean±s.e.m. for six mice. “n.s.” denotes not significantly different and “*” denotes significantly different at *P* < 0.05.

As widely reported, prostate tumorigenesis in Pten KO mice starts from low grade PIN, which progresses to high grade PIN, to localized prostate cancer, and finally to metastatic prostate cancer [19, 34, 35]. High grade PIN and focal invasive adenocarcinoma occur after 6 months of age. Treatment with RER showed no effect on the weight of prostate glands (Figure 6A). The percent of acini with low grade PIN lesions moderately increased while the percent of acini with invasive tumor cells decreased in the RER group (Figure 6B), suggesting that blocking TGF-β signaling with RER in the Pten KO mice caused moderate retardation, instead of promotion, of prostate cancer progression from high grade PIN to invasive cancer. Since tumor cell invasion into stroma starts with breakage of basement membrane, which is visualized by staining of alpha-smooth muscle actin (α-SMA) along the edge of each acinar, quantification of acini with loss of or discontinuous α-SMA staining showed that RER treatment led to a moderate reduction of acini with discontinuous α-SMA staining (Figure 6C). Consistently, staining for laminin, a component of basement membrane also showed that there were significantly higher percent of acini with positive laminin staining in the RER group than in the control group (Figure 6D). These results suggest that the TGF-β trap RER did not promote the prostate cancer progression, but moderately inhibited the progression. RER may also have a positive role in blocking metastatic potential during prostate carcinogenesis.

### RER inhibited tumor cell proliferation and Akt pathway

**Figure 7:**
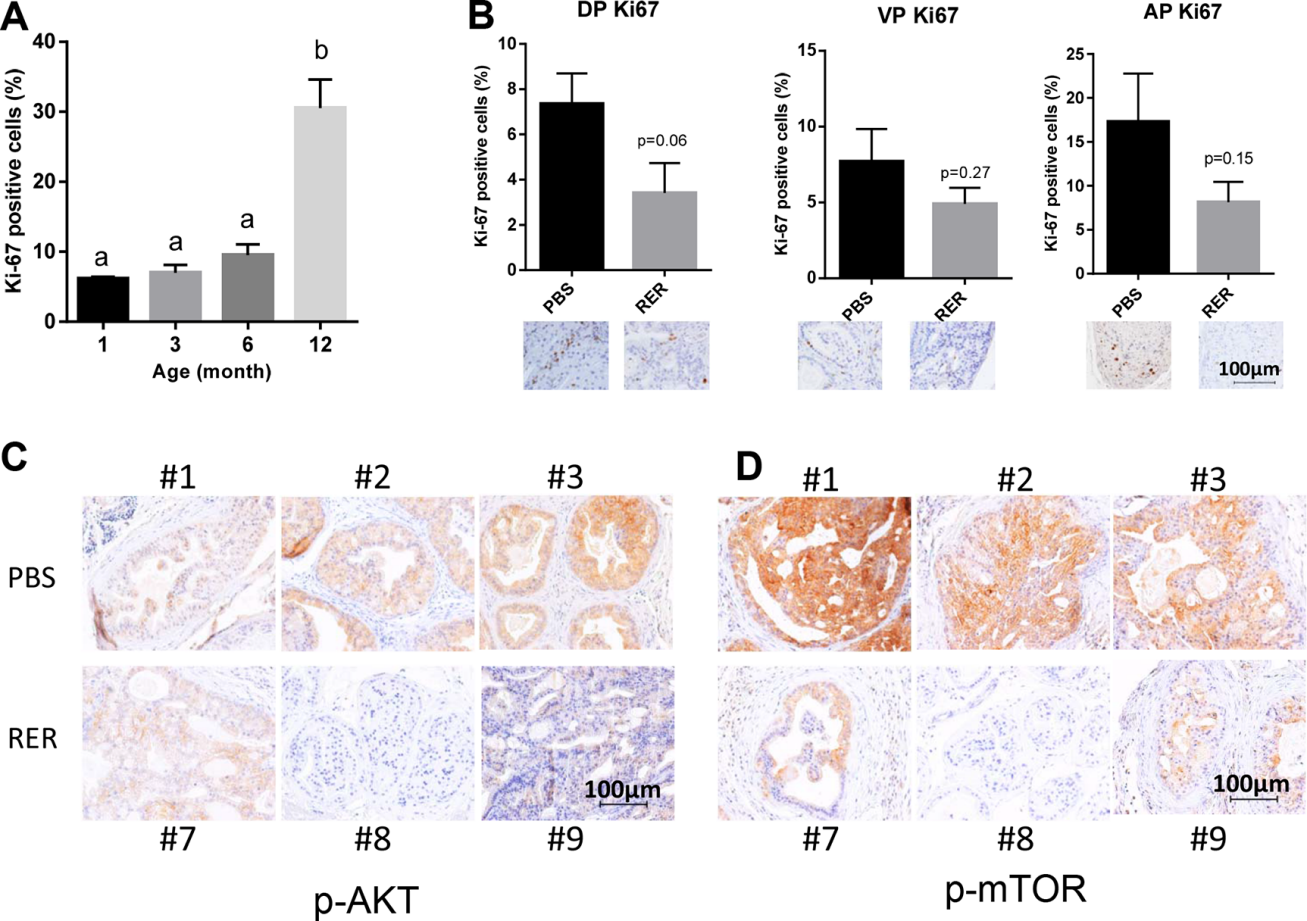
RER inhibited tumor cell proliferation and Akt pathway (**A**) Quantification of “Ki-67 positive” staining epithelial cells of DP at different age stages of Pten KO mice. “Ki-67 positive” and negative cells were counted in a total of about five hundred cells in five high power fields (HPFs) for each slide. Data presented are mean ± SEM of percent “Ki-67 positive” cells from the five HPFs for each mouse. The bars with a different letter are significantly (*P* < 0.05) different from one another with one-way ANOVA and Tukey-Kramer test. (**B**) Quantification of Ki-67 staining of DP, VP and AP lobes illustrates decreasing proliferation index in RER-treated group compared with PBS control. Ki-67-positive cells and negative cells were counted in five HPFs and expressed as percent Ki-67 positive cells. Error bars represent mean±SEM. *P* values were obtained with 2-tailed Student *t* tests. (**C**) and (**D**) IHC analysis of p-AKT and p-mTOR in three representative DP glands of each treatment group.

Since tumor progression is associated with increased tumor cell proliferation and yet autocrine TGF-β inhibited prostate cancer proliferation *in vitro*, we next examined how RER might affect tumor cell proliferation *in vivo*. In a preliminary study, we found that the frequency of the cells stained positive for the proliferation indicator Ki67 increased with the age-dependent progression of prostate cancer in the DP of Pten KO mice (Figure 7A), which is functionally most similar to human prostate gland in comparison to the AP and VP glands. Interestingly, the frequency of Ki67-positive cells was significantly decreased in the DP at *P* < 0.1 and moderately decreased in VP and AP of the RER-treated mice in comparison to the control mice (Figure 7B). Because constitutive activation of the PI3K/AKT/mTOR pathway is a major mechanism driving prostate tumorigenesis in the Pten KO mice and TGF-β signaling is known to also activate this pathway directly [30, 31] or indirectly via its regulation of other secreted factors, we next investigated whether blocking TGF-β signaling pathway with RER attenuated AKT/mTOR pathway. IHC staining with an antibody against the activated phospho-Akt^Ser473^ showed that RER-treated DP glands had lower staining intensity than the control DP glands (Figure 7C). Consistently, the staining intensity of phospho-mTOR^Ser2448^, a downstream mediator of AKT activity [32], was also much lower in the RER-treated DP glands than the control DP glands (Figure 7D). These results suggest that TGF-β acts as a growth promoter, instead of a growth inhibitor, by enhancing the AKT/mTOR pathway in the prostate gland of the Pten KO mice at the relatively early/intermediate stage of tumorigenesis.

## DISCUSSION

While a number of signal transduction pathways such as PI3K/Akt, Wnt, hedgehog, are known to drive prostate cancer progression, the TGF-β signaling pathway has been shown to be tumor suppressive in the prostate, where it inhibits proliferation and induces apoptosis [5]. However, as normal epithelial cells are transformed to tumor cells, they develop mechanisms to evade TGF-β induced-tumor suppressive activity. Once this happens, they respond to this cytokine to facilitate tumor progression [39–41]. Thus, instead of causing cell cycle arrest and/or apoptosis, TGF-β induces epithelial to mesenchymal transition (EMT), a process that facilitates migration, invasion and metastasis [42]. TGF-β also mediates the production of mitogenic growth factors, which stimulate tumor cell proliferation and survival [43], and stimulates the conversion of CD4^+^CD25^-^ T cells to CD4^+^CD25^+^Foxp3^+^ regulatory T-cells, which suppresses host immune surveillance [16, 44].

While treatment with soluble betaglycan and neutralizing antibodies have been shown to be effective in attenuating attributes of advanced PCa, namely growth and angiogenesis of tumors formed by AR negative human PCa cells [17] and suppression of host immune surveillance by regulatory T-cells [18], no studies have systematically evaluated the effects of TGF-β inhibition as prostate cancer develops. We therefore investigated this using transgenic mice bearing a prostate specific KO of the tumor suppressor Pten, which are immune-competent and develop PCa in a temporal-spatial manner that closely recapitulates human disease [19]. We furthermore used a novel inhibitor, RER, rather than an existing inhibitor such as a TGF-beta type I receptor kinase inhibitor or TGF-beta neutralizing antibody, owing to the potential limitations of these inhibitors, such as off-target activity or limited antagonistic potency. We showed as part of this study that RER derives its near picomolar antagonistic potency by virtue of its three component binding domains, which once bound to TGF-β, disassociates very slowly and completely block its ability to bind the high affinity receptor, TbRII. While comparative studies were not performed, one potential benefit of RER over the neutralizing antibody 1D11 used in this study is that it may be capable of competing against the endogenous receptor complexes for binding TGF-βs at the low tissue concentrations that we detected, whereas the antibody, due to it low nanomolar antagonistic potency [39], may not. RER’s component binding domains, including the TbRII extracellular domain and the BG endoglin domain, are both known to be highly specific for the TGF-βs, and thus one further advantage of RER should be its high specificity for binding and neutralizing signaling activity of TGF-βs, but not other TGF-β family signaling proteins. Furthermore, the relatively smaller size of RER in comparison to the neutralizing antibody might allow RER to penetrate the extracellular matrix and get closer to cell surface easier than the neutralizing antibody and consequently might allow RER to compete with the cell surface TGF-β receptors for TGF-β ligands more effectively than the antibody. On the other hand, RER may not be as stable as other TGF-β inhibitors *in vivo* as discussed below.

Our data showed that blocking TGF-β signaling in Pten KO mice at the intermediate stage of tumorigenesis moderately inhibited the progression to invasive cancer suggesting that at the age of 6–8 months of the Pten KO mice, TGF-β signaling was already acting as a tumor promoter. Prostate specific *Pten* deletion results in prostate intraepithelial neoplasia (PIN) which can progress to high-grade PIN lesion and eventually adenocarcinoma. Bjerke and co-workers found that inactivation of TGF-β signaling by the deletion of the TGF-β type II receptor gene in combination with *Pten* KO led to enhanced malignancy [40], suggesting that TGF-β signaling in the prostate epithelial cells has tumor suppressor function in Pten KO mice during prostate cancer development. In our preliminary study, we found that TGF-β1/2/3 was upregulated in the Pten KO mice with age (Supplementary Figure S8). Interestingly, increased TGF-β is mostly in stromal area. Thus, while TGF-β signaling in the prostate epithelial cells of the Pten KO mice may be tumor suppressive, its signaling in the prostate stromal cells is likely to be tumor promoting. This speculation is consistent with our previous finding that blockade of TGF-β signaling in prostate stromal cells led to a reduction of various pro-tumorigenic factors secreted by the stromal cells and significantly attenuated their ability to promote tumor growth of xenografted prostate cancer cells [46]. Yang and co-workers also reported that stromal TGF-β signaling induces AR activation in prostate cancer [43]. Thus, while it is possible that the administered RER might neutralize TGF-β signaling in both stromal and epithelial cells in the prostate, our results appear to indicate that RER might have blocked TGF-β signaling much more severely in the stromal cells than in the epithelial cells resulting in a moderate blockade of tumor progression by reducing various growth factors produced by stromal cells as we have reported previously [41]. Clearly, further studies are needed to ascertain that it is the stromal cells that mediate the tumor promoting activity of TGF-β. Furthermore it will be important to determine how early during the tumorigenesis TGF-β inhibitors show tumor-inhibitory activity and whether they show tumor-promoting activity at all. The fact that RER treatment reduced the levels of the phosphorylated AKT and mTOR in tumor cells in our study and that TGF-β was shown to rapidly activate AKT by others [42–45] suggest that RER might have also neutralized TGF-β signaling in the prostate epithelial cells. Alternatively, it is possible that blockade of stromal TGF-β signaling and consequent reduction of growth factors from the stroma might have caused the observed reduction of phosphorylation of AKT and mTOR. Thus, future studies should also focus on biomarkers in both prostate stromal cells and epithelial cells that indicate when treatment with TGF-β inhibitors are safe and beneficial during early prostate tumorigenesis for the blockade of tumor growth and metastasis. These studies are necessary for the development of TGF-β inhibitors as novel therapeutics for patients with relatively early-stage prostate cancer.

While RER showed more potent antagonistic activity than other TGF-β inhibitors in our study in blocking TGF-β signaling *in vitro*, its effects on its target gene expression and Smad2/3 phosphorylation and tumorigenic properties *in vivo* were relatively modest. Thus, while the primary objective for our *in vivo* study was to determine how blockade of TGF-β signaling might alter tumor progression at a relatively early stage utilizing the novel TGF-β inhibitor RER, a question for our future research is how RER compares with other TGF-β inhibitors in blocking tumor progression. The modest *in vivo* activity of RER could be due to a modest TGF-βactivity in the model system or a bioavailability issue with RER. We found that RER was readily detectable in liver and kidney suggesting that it might be cleared in these two organs. Interestingly, we found RER treatment altered TGF-β-regulated targets in the liver, but not the kidney suggesting that it was active in the liver and might be inactivated in the kidney. Our future studies will be focused on detailed analyses of pharmacokinetics and pharmacodynamics of RER in comparison with other TGF-β inhibitors.

In summary, our study indicates for the first time that inhibition of TGF-β signaling pathway by the systemic administration of a novel TGF-β ligand trap RER moderately suppressed the progression of relatively early/intermediate stage tumorigenesis in the prostate gland of the Pten KO mice. The inhibitory activity of RER is apparently mediated by the inhibition of TGF-β-induced tumor cell proliferation supported by AKT/mTOR pathway and tumor cell invasion.

## MATERIALS AND METHODS

### Ethics statement

All animal experiments were conducted following appropriate guidelines. They were approved by the Institutional Animal Care and Use Committee and monitored by the Department of Laboratory Animal Resources at the University of Texas Health Science Center at San Antonio.

### Chemicals and proteins

The TβRII extracellular domain (RII) and the betaglycan endoglin domain (BG_E_) were produced in bacteria and renatured and purified as previously described [46, 21]. The ER and RER receptor traps were produced by transient transfection of HEK293F cells grown in suspension in Freestyle 293 medium at 8% CO_2_, 80% humidity, and rotating at 80 rpm (Infors HT, Laurel, MD). The proteins were purified from the conditioned medium seven days post-transfection using a combination of metal affinity and size exclusion chromatography. Additional details regarding the expression and purification are provided as Supplementary Figure S3. The TGF-β pan-neutralizing antibody 1D11 was purchased from BioXCell (BioXCell, West Lebanon, NH). HTS466284 (HTS), an ATP competitive inhibitor of TβRI kinase [47, 48], was synthesized by the Chemical Synthesis Core of Vanderbilt University.

### Cell culture

Human prostate cancer cell line PacMetUT1 was isolated from the lymph node metastasis of a 57-year old prostate cancer patient at our university [49]. PC-3 and DU145 were purchased from the American Type Culture Collection (ATCC, Manassas, VA, USA). PacMetUT1 and DU145 cells were cultured in McCoy’s 5A medium supplemented with pyruvate, amino acids, nutrients and 10% fetal bovine serum (FBS), as described [50]. HEK293 FreeStyle cells (HEK293F, Invitrogen, Carlsbad, CA) were maintained in FreeStyle 293 Medium (Invitrogen, Carlsbad, CA) supplemented with 1% Penicillin/Streptomycin. Mink lung epithelial (Mv1Lu) cells were obtained from Prof. Dan Rifkin and were maintained in Dulbecco’s Modified Eagle’s Medium supplemented with 10% FBS and 1% penicillin/streptomycin. PacMetUT1, DU145, and Mv1Lu cells were maintained at 37°C in a 5% CO_2_ humidified incubator, while the HEK293F cells were maintained at 37°C in an 8% CO_2_humidified incubator-shaker.

### TGF-β luciferase reporter and Smad phosphorylation assays

Mv1Lu cells containing a PAI-1-luciferase reporter gene [51] were utilized for the TGF-β reporter assays. Cells were treated for 16 hours at 37^°^C with 20 pM TGF-β1, -β2, or –β3 and varying concentrations of ER or RER receptor traps. Cells were then lysed and luciferase activity was measured with a luminometer (Promega Corp.). Luciferase responses were normalized to that of non-inhibited controls and the dose response curves were then fitted to a standard equation for inhibition with variable slope using Graphpad Prism (Graphpad Software). TGF-β induced phosphorylation of Smad2 and Smad3 was assessed by treating exponentially-growing MCF-10A human breast epithelial cells with 2 pM TGF-β1 and 0.01 – 10 nM ER or RER. Western blotting was performed as described below.

### Cell proliferation assay

Cells were plated in a 96-well plate at 2,000 cells/well in triplicate. After treatment with TGF-β1, RER, HTS, or 1D11 individually or in combination for 4 days, MTT (3-(4,5-dimethylthiazol-2-yl)-2,5-diphenyltetrazolium bromide at 2 mg/ml in phosphate-buffered saline, PBS) solution was added at 50 μl/well, and cells were incubated at a 37°C cell culture incubator for 2 hours. For dissolving the blue-colored formazon product, dimethyl sulfoxide (100 μl) was added into each well after the medium was removed. The plate was gently shaken on a shaker for 10 min. The absorbance was measured at 595 nm with a Microplate Reader (BioTek Instrument, Winooski, VT).

### Cell migration assay

Cell migration assays were performed in 24-well Transwells with 8-μm pore polycarbonate membranes (BD Biosciences). Cells at a density of 40,000 cells/well in a serum-free medium with or without treatment were seeded in the upper insert in triplicate. Complete medium with or without treatment was added in the lower chamber. After 14 h for PacMetUT1 and PC-3, 8 h for DU145, cells that did not migrate across the membrane were removed with a cotton swab and migrated cells were stained with the Hema 3 Stain 18 kit (Fisher Scientific, Waltham, MA) according to the manufacturer’s protocol. Migrated cells were counted under a microscope with 100× magnification.

### Western blot analysis

Exponentially growing cells were harvested and lysed in Laemmli buffer with a cocktail of protease inhibitor. The total protein concentration was quantified using the bicinchoninic acid protein assay (Thermo Scientific, Rockford, IL). Equal amounts of total protein were resolved by SDS-PAGE and transferred to a nitrocellulose membrane under a constant voltage. Membranes were blocked with 5% nonfat dried milk in TBST (100 mM Tris-HCI pH 8.0, 150 mM NaCl, 0.05% Treen-20). Primary antibodies and secondary antibodies were diluted in TBST with 3% milk or BSA and applied with a triplicate washing step in between. Antibodies were purchased from the following sources: Smad2, phospho-Smad2, phospho-Smad3, Akt, phospho–Akt, Erk, phosphor-Erk, Snail, PTEN from Cell Signaling Technology (Danvers, MA); Smad2/3, Vimentin from BD Transduction Laboratories (San Jose, CA); E-Cadherin from Santa Cruz Biotechnology (Santa Cruz, CA); and TβRII from R&D (Cambridge, MA). Proteins were detected by chemiluminescence procedures with ECL reagents (Millipore).

### Animal Study

Pten conditional knockout (*Pten^flx/flx^*, *Pb-Cre4; Pten^flx/+^*, *Pb-Cre4*) mice were created by Dr. Haojie Huang in Mayo Clinic. *Pten^flx/flx^*, *Pb-Cre4* male mice were mated with *Pten^flx/flx^*, *Pb-Cre4(-)* female mice to generate a cohort of *Pten^flx/flx^*, *Pb-Cre4* male mice for experiments. Mouse tail DNA was used for PCR-based genotyping using MyTaq™ Extract-PCR Kit (Bioline, London, UK). For the treatment with TGF-β ligand trap RER, we used 12 6–8 month-old male mice and grouped them for PBS (control) or RER treatment with 6 mice in each group. RER was diluted in sterile 1xPBS and were intraperitoneally (i.p.) injected at a dose of 50 μg/mouse/day for 30days. An equal volume of 1× PBS were i.p. injected in control mice as a placebo.

### RNA extraction and quantitative real-time PCR

RNA was isolated from dorsolateral and ventral prostate of *Pten^flx/flx^*. *Pb-Cre4* male mice using RNA Mini Spin Column of EnzyMax LLC (Lexington, KY) according to the manufacturer’s instructions. The extracted RNA was shredded by EZshredder column of EnzyMax LLC (Lexington, KY) to remove genomic DNA contamination. Total RNA (2 μg) was reverse-transcribed into cDNA using random primers and M-MLV reverse transcriptase from Invitrogen Life Technology (Grand Island, NY). Quantitative real-time PCR (qRT-PCR) was performed using Power SYBR Green PCR Mix from Life Technologies. All primers used in this study were designed by Primer Blast of NCBI and synthesized by Integrated DNA Technologies (Coralville, IA). Primer pair specificity was determined by generation of a single peak for dissociation curve (melting curve) at the end of RT-PCR cycling program. Primer sequences for E-cadherin are (Forward:GGCTGGACCGAGAGAGTTACC. Reverse: CACTTTGAGTGTGGCGATCC) and for vimentin are (Forward: GACCAGAGATGGACAGGTGAT. Reverse: CGTCTTTTGGGGTGTCAGTTG).

### Immunohistochemistry (IHC) assay

Tissue sections of formalin-fixed and paraffin-embedded ventral, dorsolateral, and anterior prostate lobes of mouse prostate, and their liver and kidney were cut at a 4.5 μm in thickness with a LEICA RM2255 microtome and dried at room temperature for 24 h. Sections were heated to 75°C for 15 min and then rehydrated through xylene and graded ethanol, incubated in a sodium citrate solution (10 mM, pH6.0) or a EDTA solution (1 mM, pH8.0) at 95°C for 15min or 30min, respectively. Sections were then blocked for endogenous peroxidase with 3% hydrogen peroxide (Thermo Fisher Scientific, Waltham, MA, USA) for 15 min at room temperature and were permeabilized and blocked in 10% goat or donkey serum for 1 hr. The primary antibodies to phospho-Smad2, phospho-Smad3, phosphor-Akt, phosphor-mTOR were purchased from Cell Signaling Technology (Danvers, MA), to cytokeratin 8 (CK8) and laminin from Abcam (Cambridge, MA), to TβRII from R&D (Cambridge, MA), to E-Cadherin from Santa Cruz Biotechnology (Santa Cruz, CA), and to Ki67 from Thermo Fisher Scientific (Waltham, MA). Primary antibodies were diluted in 1xPBS with 0.025% Triton-100 and 5% serum and incubated with the tissue sections at 4°C overnight. Sections were then incubated with a biotinylated secondary antibody (BD Pharmingen, San Diego, CA). For detection, Streptavidin-Horseradish Peroxidase and DAB Substrate Kit (BD Pharmingen, San Diego, CA) were used and the counterstain was done with hematoxylin.

### Statistical analysis

Two-tailed Student’s *t*-test was used to compare the means of two groups. One-way analysis of variance with Tukey-Kramer post hoc test was used for analyzing data when means from more than two groups were compared. Results are expressed as mean ± sem. *P* < 0.05 was considered to be statistically significant.

## SUPPLEMENTARY TABLES AND FIGURES


