# Correction: A novel highly potent trivalent TGF-β receptor trap inhibits early-stage tumorigenesis and tumor cell invasion in murine Pten-deficient prostate glands

**DOI:** 10.18632/oncotarget.20370

**Published:** 2017-08-21

**Authors:** Tai Qin, Lindsey Barron, Lu Xia, Haojie Huang, Maria M. Villarreal, John Zwaagstra, Cathy Collins, Junhua Yang, Christian Zwieb, Ravindra Kodali, Cynthia S. Hinck, Sun Kyung Kim, Robert L. Reddick, Chang Shu, Maureen D. O’Connor-McCourt, Andrew P. Hinck, Lu-Zhe Sun

Copyright: Qin et al. This is an open-access article distributed under the terms of the Creative Commons Attribution License 3.0 (CC BY 3.0), which permits unrestricted use, distribution, and reproduction in any medium, provided the original author and source are credited.

**Present:** The grant funding information is incomplete.

**Correct:** Additional grant information appears below. The authors sincerely apologize for this omission..

Original article: Oncotarget. 2016; 7:86087-86102. https://doi.org/10.18632/oncotarget.13343

FUNDING

M.M.V. and S.K.K. were supported by Cancer Research Training Program grants RP101491 and RP140105, respectively, from Cancer Prevention and Research Institute of Texas (CPRIT).

