# Supplementary material for: A novel highly potent trivalent TGF-β receptor trap inhibits early-stage tumorigenesis and tumor cell invasion in murine Pten-deficient prostate glands
Source: Oncotarget. 2016 Nov 14;7(52):86087–102. doi: 10.18632/oncotarget.13343 (PMC5349899; doi:10.18632/oncotarget.13343)
Supplement: Supplementary file 1 [file oncotarget-07-86087-s001.pdf]

A novel highly potent trivalent TGF-β receptor trap inhibits early-stage tumorigenesis and tumor cell invasion in murine Pten-deficient prostate glands

Supplementary Materials

Design of ER and RER receptor traps

Structural information is available for TGF-βs bound to TβRII (RII) [1-3], but not betaglycan endoglin domain (BG_E) bound to TGF-β – hence the challenge of designing trap constructs comprised of BG and RII involves choosing linkers that are long enough so as to not restrict binding, but not so long that the beneficial effects of multivalent binding are unattained.

When we designed BG -RII (ER), we noted from the TGF-β:TβRII structure [1–3] that the TβRII N-terminus extends toward the center of the TGF-β homodimer where BG must be bound (Supplementary Figure S1). We reasoned that a linker of 27 amino acid residues should be sufficient since once extended it could reach distances of up 94.5 Å (3.5 Å/residue for an extended polypeptide chain times 27 residues), which is longer than the longest dimension of a TGF-β homodimer (78 Å; Supplementary Figure S1). We did not, however, strictly introduce a linker of this length, but also took into account expected regions of structural disorder in the terminal regions of the receptors – accordingly, we choose an artificial linker with just 2 residues since in the constructs used, BG included what we believed to be 25 structurally disordered residues, while TβRII included none (Supplementary Figure S2).

We applied similar logic to design the linker to expand ER to RER, although in this case a longer linker (32 residues) was used since the structure of the TGF-β receptor complex showed that the TβRII C-terminus extended away from the center of the TGF-β homodimer, rather than toward it (Supplementary Figure S1). Since there were 9 structurally disordered residues in TβRII and 5 in BG_E, we introduced an artificial linker 18 residues in length (Supplementary Figure S3). We tried to include a sufficient number of proline, serine, and glycine residues in the artificial linker, which are known to be enriched in intrinsically disordered proteins [4], while at the same time excluding residues or patterns of residues recognized by matrix metalloproteinases [5].

Construction of ER and RER expression vectors

A CMV-based expression construct for the ER receptor trap was generated by first replacing the BamHI restriction site following the coding sequence for the rat serum albumin signal peptide in plasmid pcDNA3.1+-GS [6] with the sequence for a NotI restriction site. The coding cassette for the rat BG -human TβRII receptor fusion with a C-terminal histidine tag in the bacterial expression vector pET17b [7] was then PCR amplified with a sense primer that had a NotI restriction site in the 5′ overhang and was complementary beginning with G24 of rat betaglycan and an antisense primer that had an XbaI restriction site in the 5′ overhang region and was complementary to the C-terminal portion of the previous ER construct, which extended to D159 of human TβRII, followed by six histidine residues, and a stop codon. The PCR product was then digested with NotI and XbaI and inserted into similarly digested NotI-modified pcDNA3.1+-GS to yield the CMV-based ER expression vector shown in Figure 1C.

A CMV-based expression vector for the RER receptor trap was generated by first PCR amplifying the coding sequence for human TβRII in three steps: In all three steps, the sense primer had a NotI restriction site in the 5′ overhang region and was complementary beginning with N42 of human TβRII, while the antisense primer was sequentially shifted so as to add the coding sequence for a GLGPVESSPGHGLDT linker and a NotI restriction site following D159 of human TβRII. The PCR product was then digested with NotI and inserted into NotI-digested, calf intestinal alkaline phosphatase-treated CMV-based ER expression vector. Clones containing inserts with the correct orientation were identified by DNA sequencing. A diagram of the CMV-based RER expression vector is shown in Figure 1D.

Oligonucleotides used and the complete amino acid sequences of the ER and RER constructs are provided as Supplementary Table S1, Supplementary Figures S2-S3).

Expression and purification of ER and RER

The ER and RER receptor traps were produced by transient transfection of HEK293F cells grown in suspension in Freestyle 293 medium at 8% CO , 80% humidity, and rotating at 80 rpm (Infors HT, Laurel, MD). For each protein, cells were grown to a density of 1.25 × 10 cells mL in 1 L of medium contained in a 5 L Optimum Growth Flask (Thomson Instruments). Cells were harvested by centrifugation, resuspended in 500

mL fresh medium, and incubated with 1.5 mg of cesium chloride gradient purified plasmid DNA, followed by the addition 4.5 mL 1 mg mL polyethylenmine (Polysciences, Warrington, PA). 24 hours later, the cells were diluted with 500 mL medium followed by the addition of valproic acid (VPA, Sigma-Aldrich, St. Louis, MO) to a final concentration of 2.2 mM. Conditioned media were collected by centrifugation six days after the transfection, filtered, and stored at −20 C.

The conditioned media were diluted 1:1 with column buffer (25 mM sodium phosphate buffer, pH 8.0, 150 mM NaCl, 8 mM imidazole, 1 mM nickel sulfate) and adjusted to pH 8.0 by the addition of solid Tris base. Nickel sulfate was added to achieve a final concentration of 1 mM. Protein was bound to Ni++-loaded chelating sepharose column (GE Lifesciences) that had been equilibrated with column buffer, and after washing with column buffer, was eluted by application of a linear imidazole gradient from 8 to 500 mM. Protein containing fractions were identified by SDS-PAGE, pooled, and dialyzed against 25 mM Hepes pH 8.0, 50 mM NaCl, and 0.05% sodium azide. For further purification, samples were subjected to gel filtration chromatography on a Superdex 200 (GE) column in the Hepes buffer used for dialysis. Fractions containing pure protein were pooled and stored at -20◦C. Concentrations of the purified ER or RER samples were determined from their UV absorbance at 280 nM and their calculated extinction coefficients, 40930 and 49900 M^{-1} cm^{-1}, respectively. Deglyosylated samples for SDS-PAGE were generated by incubating 2 μg of purified ER or RER with 2 μg peptide-N4-(N-acetyl-beta-glucosaminyl) asparagine amidase (PNGaseF) for 12 h at 37°C in 2.5 M urea, 25 mM sodium phosphate, 50 mM NaCl, pH 8.0.

SPR and ITC methods

Surface plasmon resonance (SPR) and isothermal titration calorimetry (ITC) were used to assess the binding of the ER and RER receptor traps to the TGF-βs. SPR was also used to assess binding of ER- or RER-bound TGF-β3 to immobilized TβRII. SPR binding studies were performed using a BIAcore 3000 instrument with streptavidin-coated carboxy methyl dextran (CM5) sensor chips (GE Lifesciences) with TGF-β2, TGF-β3, or TβRII captured at a surface density of 50–80 resonance units (RU). We accomplished this by expressing the mature domain of human TGF-β2, human TGF-β3, or the human TβRII ectdomain with a 15 amino acid avitag [8] followed by an EY dipeptide attached to either the N- (TGF-β2 and TGF-β3) or C-terminus (TβRII) in bacteria [8]. Insoluble avi-tagged TGF-β2, TGF-β3, or TβRII was reconstituted in urea, refolded, and purified to homogeneity using procedures previously described [9]. Avi-tagged TGF-β2 and TGF-β3 were then complexed with betaglycan in 10 mM bicine at pH 8.0 and biotinylated by incubating with a catalytic amount of bacterially expressed BirA recombinase, biotin, and ATP at 37°C for 2 hr as described [9]. Avi-tagged TβRII was biotinylated in the same manner, although it was unnecessary to form a complex since unlike the TGF-βs, TβRII is soluble in the absence of any of its partners. Biotinylated avi-tagged TGF-β2, TGF-β3, or TβRII were then bound to a C4 reverse phase column equilibrated with 94.9% water/5% acetonitrile/0.1% triflouroacetic acid and eluted with a linear acetonitrile gradient. Attachment of a single biotin to each protein chain was confirmed by measuring the intact mass of the biotinylated, purified proteins using electrospray ionization-time of flight mass spectrometry (Agilent).

SPR binding assays were performed by injecting either a) two-fold serial dilutions of ER or RER in duplicate in HBS-EP buffer (GE Healthcare) at a flow rate of 100 μL min over the TGF-β2 or TGF-β3 surface, or b) two-fold serial dilutions of TGF-β3:ER or TGF-β3:RER complexes over the TβRII surfaces. TGF-β3:ER or TGF-β3:RER complexes were prepared by mixing a two-fold excess of ER or RER relative to TGF-β3 and by the applying the mixtures to Superdex 200 (GE) column equilibrated in HBS. Surfaces were regenerated by a brief injection of 4 M guanidine hydrochloride adjusted to a pH 4 with acetic acid (10 s contact time at a flow rate of 100 μL min^{-1}). Baseline correction was performed by double referencing [10]. Kinetic analyses were performed by global fitting with a simple 1:1 model using the Biaevaluation software (GE Lifesciences). Standard errors were obtained from the variation in the fitted parameters.

Isothermal titration calorimetry (ITC) data was generated using a Microcal PEAQ-ITC instrument (Malvern Instruments). ER and RER receptor trap proteins were exhaustively dialyzed against 10 mM Na_HPO pH 7.4. Prior to transfer to the calorimeter syringe, they were concentrated to 50–110 μM and then (3-(3-cholamidopropyl) dimethylammonio)-1-propanesulfonate) (CHAPS) was added to a final concentration of 30 mM. TGF-β2 was dialyzed into 100 mM acetic acid, lyophilized, and then resuspended in the dialysis buffer (10 mM NaH PO pH 7.4) supplemented with 30 mM CHAPS at a concentration of 5–8 μM before being placed in the calorimeter cell. Titrations were performed at 37°C. 20 2 uL injections were performed with a injection duration of 4 sec, a spacing of 110 sec, and a reference power of 6. Data analysis was performed using the PEAQ-ITC software provided with the instrument.

ER and RER binding properties

To understand the improved antagonistic potency of RER compared to ER, kinetic SPR experiments were performed in which ER or RER, or their component binding domains, BG (E), and TβRII (RII), were injected in HEPES buffer saline (HBS) over immobilized TGF-β2 or TGF-β3 for a relatively short period (150 s) at high flow rate (100 μL min^{-1}), followed by a longer disassociation

period. The sensorgrams obtained for injection of RII and BG over the TGF-β2 and TGF-β3 were similar to those reported earlier (Supplementary Figure S4A–S4D) [11, 12]. RII exhibited a clear preference for binding TGF-β3 over TGF-β2 and associated and disassociated with rapid kinetics, while BG bound TGF-β2 and TGF-β3 comparably and associated and disassociated with rates that were considerably slower. The sensorgrams were analyzed by performing a global fit to a 1:1 binding model and the fitted rate constants, and other parameters, are listed in Supplementary Table S3 (response for RII binding to TGF-β2 was too weak, and thus no parameters are listed for this interaction).

The sensorgrams obtained for injection of ER over the TGF-β2 and TGF-β3 surfaces differ from those of RII and BG_E in that the disassociation rates are much slower than either of the component domains, especially for TGF-β3, but also for TGF-β2 (Supplementary Figure S4A–S4F). The fitted dissociation rate constants, k_d, which were also obtained by performing a global fit of the data to a 1:1 binding model, were found to be roughly 1 and 3 orders of magnitude slower for the ER trap compared to BG for binding TGF-β2 and TGF-β3, respectively (Supplementary Table S3). The corresponding association rate constants, k_a, in contrast were only 2–3 fold slower than that of BG_E, and thus the corresponding affinities were roughly 20- and 2000-fold greater than that of BG_E (K_D 1.4 and 30.6 nM for TGF-β2 binding ER and BG_E, respectively; K_D 50.5 pM and 94600 pM for TGF-β3 binding ER and BG_E, respectively). The observed attenuation of k_d and corresponding increase in affinities can be attributed to the additive effects of multivalent binding since the observed increase was greater for TGF-β3, which has relatively high affinity for both of its component binding domains, compared to TGF-β2, which has relatively high affinity for BG_E, but not RII.

The sensorgrams obtained for injection of RER over the TGF-β2 and TGF-β3 surfaces are comparable to those of ER, though as shown by global fitting to a 1:1 model as before, the addition of the N-terminal RII domain, leads to a further attenuation of k_d, with k_d for TGF-β2 and TGF-β3 being 1.7- and 2.1-fold slower, respectively. This leads to an approximate further two-fold increase in affinity as the k_a values are largely unchanged (K_D 0.82 nM and 1.4 nM for TGF-β2 binding to RER and ER, respectively; K_D 24 pM and 51 pM for TGF-β3 binding to RER and ER, respectively).

ER and RER binding stoichiometry and blockage of TβRII binding

The K_D values determined for ER binding TGF-β2 and TGF-β3 are roughly consistent with the antagonistic potencies measured by the luciferase assay, with K_Ds of 1440 and 51 pM, respectively, and IC_{50}s of 1200 and 20 pM, respectively. The K_D values determined for RER binding to TGF-β2 and TGF-β3, are in contrast somewhat higher than the antagonistic potencies measured by the luciferase assay, with K_Ds of 823 and 24 pM, respectively and IC_{50}s of 70 and 3.3 pM, respectively. This suggests that other factors might be contributing to RER's potency, and accordingly we investigated the stoichiometry with which the traps bind TGF-β and whether they blocked TβRII binding or not.

The stoichiometry with which ER and RER bind TGF-β2 was assessed using ITC, with TGF-β2 at a concentration of 5 – 10 μM in the calorimetry cell and ER or RER in the syringe at a concentration of 50–100 μM (Table 3). The TGF-β2 and traps were both prepared in phosphate buffer at neutral pH (pH 7.4), although, it was necessary to also include 30 mM CHAPS in the buffer so as to solubilize TGF-β2, which along with the other TGF-β isoforms are known to be poorly soluble between pH 5 and 9 [13]. Though it was necessary to include CHAPS in the buffer solubilize TGF-β2, we nonetheless knew that this would weaken binding as accompanying SPR experiments (not shown) demonstrated that CHAPS weakened BG_E and TβRII binding by TGF-β3 by 25-fold and 2-fold, respectively.

The raw and integrated ITC data for binding of ER and RER to TGF-β2 in the presence of 30 mM CHAPS are shown in Supplementary Figures 5A–5B and 5C–5D, respectively. There was well-defined curvature in both titrations, enabling reliable fitting of the integrated heats to obtain a K_D of 215 nM and a stoichiometry of 1.05 ± 0.07 for ER binding to TGF-β2 and a K_D of 128 nM and a stoichiometry of 1.10 ± 0.05 for RER binding to TGF-β2 (Supplementary Table S4). The ITC-derived affinities measured in 30 mM CHAPS are in the range expected assuming an approximate 75-fold and 225-fold weakening relative to the SPR derived affinities in the absence of CHAPS for ER and RER, respectively. The ITC-derived stoichiometries are also consistent with expectations – for RER, all three domains of RER are expected to bind, leaving no available sites for another RER molecule to bind – for ER, both domains are expected to bind, and while one site for TβRII should remain unoccupied and thus available for binding of a second molecule of ER through its RII domain, this is not expected under the concentrations used in the ITC experiment due to the weak intrinsic affinity of RII for TGF-β2 (Supplementary Figure S4A).

To investigate ability of ER and RER to block TβRII binding, a competition SPR experiment was performed in which pre-formed ER:TGF-β3 or RER:TGF-β3 complexes were injected over avitag-immobilized TβRII. The pre-formed ER:TGF-β3 or RER:TGF-β3 complexes were prepared by adding 1.1 molar equivalents of ER or RER to TGF-β3, followed by isolation of the complexes in HBS buffer using size exclusion chromatography (isolated

complexes are shown in the insets to Figure 2G, 2H). The isolated complexes were then injected in HBS buffer at high flow rate (100 μL min^{-1}) over immobilized TβRII at concentrations comparable to or higher than the K_D for binding of TGF-β3 to TβRII (0.5, 1, 2, and 4 μM). This resulted in a robust concentration-dependent response when the ER:TGF-β3 complex was injected, but not when the RER:TGF-β3 complex was injected (Figure 2G, 2H). Though the sensorgrams for binding of the ER:TGF-β3 complex to TβRII were not fit to obtain the parameters for binding, they are nonetheless consistent with that expected for the TGF-β3 and TβRII interaction, which has a K_D in the range of 200–400 nM. In summary, the ITC and SPR competition demonstrate the ER and RER bind TGF-βs with 1:1 stoichiometry, and while RER completely blocks the ability of trap-bound TGF-β to bind to TβRII, ER does not.

RER was distributed over multiple organs after i.p. injection in *Pten* conditional knockout mice

We used 6-8 month-old prostate specific knockout mice (Pten KO) to determine the effect of RER on prostate tumor progression. Six homozygous Pten KO male mice were i.p. injected with PBS as the control group while the other six mice were injected with RER at 50 μg/mouse/day for 30 days. After the termination of the experiment, we first examined the distribution of RER in the circulation and organs including prostate gland, liver and kidney. Immunoblotting with an anti-TβRII antibody detected a protein band in the serum with slightly slower mobility than the purified RER (Supplementary Figure S7A). Given the fact that this protein was only detected in the serum samples from four of the RER-treated mice, we believe it is the injected RER and the change of mobility might be due to aberrant modification of RER and/or the presence of serum in the samples that altered the mobility of RER in gel electrophoresis. Using IHC with the anti-TβRII antibody, we also observed varying intensities and patterns of staining in various tissues, and the staining was mostly heavier in the tissues of the RER-treated mice as illustrated with the staining in the anterior prostate glands (AP) from the six RER-treated mice labeled #7 to #12 in Supplementary Figure S7B when compared with those from the six control mice labeled #1 to #6. Supplementary Figure S7C shows representative IHC staining of tissues including the dorsolateral and ventral prostate glands (DP and VP respectively), the liver, and the kidney. These results indicate wide distribution of the injected RER in various tissues *in vivo*.

REFERENCES

1. Groppe J, Hinck CS, Samavarchi-Tehrani P, Zubieta C, Schuermann JP, Taylor AB, Schwarz PM, Wrana JL, Hinck AP. Cooperative assembly of TGF-beta superfamily signaling complexes is mediated by two disparate mechanisms and distinct modes of receptor binding. Mol Cell. 2008; 29:157–168.
2. Hart PJ, Deep S, Taylor AB, Shu Z, Hinck CS, Hinck AP. Crystal structure of the human TbetaR2 ectodomain--TGF-beta3 complex. Nat Struct Biol. 2002; 9:203–208.
3. Radaev S, Zou Z, Huang T, Lafer EM, Hinck AP, Sun PD. Ternary complex of transforming growth factor-beta1 reveals isoform-specific ligand recognition and receptor recruitment in the superfamily. J Biol Chem. 2010; 285:14806–14814.
4. Campen A, Williams RM, Brown CJ, Meng J, Uversky VN, Dunker AK. TOP-IDP-scale: a new amino acid scale measuring propensity for intrinsic disorder. Protein Pept Lett. 2008; 15:956–963.
5. Kumar S, Ratnikov BI, Kazanov MD, Smith JW, Cieplak P. CleavPredict: A Platform for Reasoning about Matrix Metalloproteinases Proteolytic Events. PLoS One. 2015; 10:e0127877.
6. Zou Z, Sun PD. An improved recombinant mammalian cell expression system for human transforming growth factor-beta2 and -beta3 preparations. Protein Expr Purif. 2006; 50:9–17.
7. Verona EV, Tang Y, Millstead TK, Hinck AP, Agyin JK, Sun LZ. Expression, purification and characterization of BG(E)RII: a novel pan-TGFbeta inhibitor. Protein Eng Des Sel. 2008; 21:463–473.
8. Cull MG, Schatz PJ. Biotinylation of proteins *in vivo* and *in vitro* using small peptide tags. Methods Enzymol. 2000; 326:430–440.
9. Huang T, Hinck AP. Production, Isolation, and Structural Analysis of Ligands and Receptors of the TGF-beta Superfamily. Methods Mol Biol. 2016; 1344:63–92.
10. Rich RL, Myszka DG. Grading the commercial optical biosensor literature-Class of 2008: 'The Mighty Binders'. J Mol Recognit. 2010; 23:1–64.
11. Mendoza V, Vilchis-Landeros MM, Mendoza-Hernandez G, Huang T, Villarreal MM, Hinck AP, Lopez-Casillas F, Montiel JL. Betaglycan has two independent domains required for high affinity TGF-beta binding: proteolytic cleavage separates the domains and inactivates the neutralizing activity of the soluble receptor. Biochemistry. 2009; 48:11755–11765.
12. Huang T, David L, Mendoza V, Yang Y, Villarreal M, De K, Sun L, Fang X, Lopez-Casillas F, Wrana JL, Hinck AP. TGF-beta signalling is mediated by two autonomously functioning TbetaRI:TbetaRII pairs. EMBO J. 2011; 30:1263–1276.
13. Pellaud J, Schote U, Arvinte T, Seelig J. Conformation and self-association of human recombinant transforming growth factor-beta3 in aqueous solutions. J Biol Chem. 1999; 274:7699–7704.

Supplementary Table S1: Oligonucleotides used to construct the ER and RER expression constructs

Oligonucleotide Purpose	Sense or Antisense	Oligonucleotide sequence
Mutagenesis to introduce NotI site into pcDNA3.1+−GS	Sense	5′ CCGGTTCTGCCTTTTCTGCGGCCGCCCACCACCACCACCACCA 3′
Mutagenesis to introduce NotI site into pcDNA3.1+−GS	Antisense	5′ TGGTGGTGGTGGTGGTGGGCGGCCGCAGAAAAGGCAGAACCGG 3′
Amplification of ER expression cassette from pET17b	Sense	5′ AATGCGGCCGCTGGTCCAGAGCCTGGTG 3′
Amplification of ER expression cassette from pET17b	Antisense	5′ AATTCTAGATCAATGATGATGATGATGATGG 3′
Amplification of TβRII to insert into NotI site of NotI-modified pcDNA 3.1+−GS	Sense	5′ AATGCGGCCGCTAACGGTGCAGTCAAGTTTC 3′
Amplification of TβRII to insert into NotI site of NotI-modified pcDNA 3.1+−GS	Antisense, Round 1	5′ TTCCACAGGACCAAGGCCGTCAGGATTGCTGGTG 3′
Amplification of TβRII to insert into NotI site of NotI-modified pcDNA 3.1+−GS	Antisense, Round 2	5′ GCCATGGCCAGGTGATGATTCCACAGGACCAAG 3′
Amplification of TβRII to insert into NotI site of NotI-modified pcDNA 3.1+−GS	Antisense, Round 3	5′ AATGCGGCCGCCGTGTCCAGGCCATGGCCAGG 3′

Supplementary Table S2: Antagonistic potency (IC_{50}) of ER and RER for TGF-β1, -β2, and -β3

	TGF-β1	TGF-β2	TGF-β3
ER	14 ± 9 pM (*n* = 2)	1200 ± 300 pM (*n* = 2)	20 ± 11 pM (*n* = 2)
RER	0.51 ± 0.22 pM (*n* = 3)	70 ± 18 pM (*n* = 3)	3.3 ± 5.8 pM (*n* = 3)

Supplementary Table S3: SPR binding constants for ER or RER to TGF-β2 or TGF-β3

Surface	Analyte	k_{on}($M^{-1} s^{-1}$)	k_{off}(s^{-1})	K_D(pM)	$R_{ma×}$(RU)
TGF-β2	TβRII	n.d.	n.d.	n.d.	n.d.
TGF-β2	BG	1.52×10^{5}	4.66×10^{-3}	30.6×10^{3}	178
TGF-β2	ER	3.07×10^{5}	4.38×10^{-4}	1439	85
TGF-β2	RER	3.07×10^{5}	2.53×10^{-4}	823	55
TGF-β3	TβRII	3.99×10^{5}	1.36×10^{-1}	401×10^{3}	150
TGF-β3	BG	1.31×10^{5}	1.24×10^{-2}	94.6×10^{3}	167
TGF-β3	ER	3.91×10^{5}	1.97×10^{-5}	50.5	182
TGF-β3	RER	3.87×10^{5}	9.44×10^{-6}	24.4	153

*n.d., not determined

Supplementary Table S4: ITC binding data for ER or RER to TGF-β2

	ER	RER
TGF-β2 Concentration (μM)	8.50	5.00
Trap concentration (μM)	100	54.2
N (sites)	1.05 ± 0.068	1.10 ± 0.045
K_D (nM)	215 ± 181	128 ± 68.4
ΔH (kcal mol)	−7.07 ± 0.91	−16.4 ± 1.4
ΔG (kcal mol)	−9.11	−9.73
-TĎS (kcal mol)	−2.03	6.69

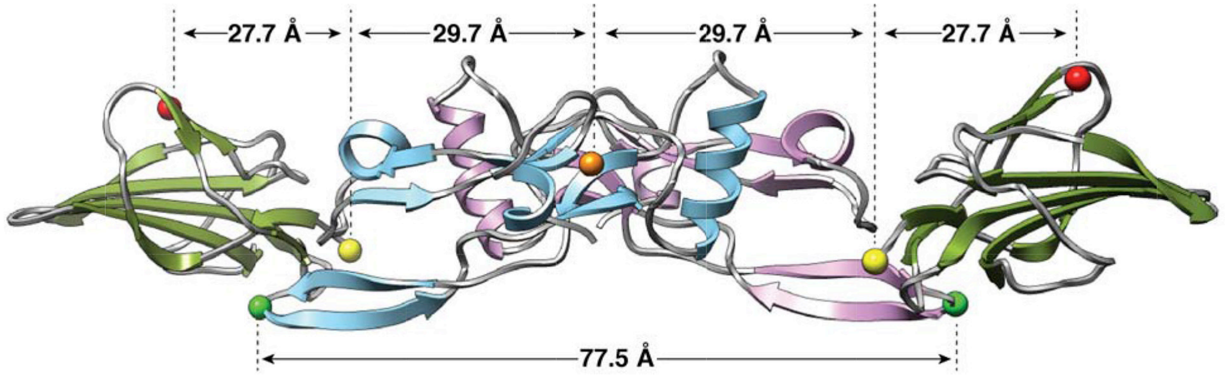

Supplementary Figure S1: Structure of TGF-β1 bound to TβRII. Structure is shown as a ribbon diagram, with the TGF-β1 monomers depicted in light blue and pink and the two bound TβRIIs depicted in olive. The center of the TGF-β1 homodimer is indicated by an orange sphere, while the last structurally ordered residue on the N- and C-terminus of TβRII (P48 and E151) is shown as either a yellow or red sphere, respectively. The Cα carbon of Arg94 on the tips of the TGF-β1 homodimer is marked by a green sphere. Structure is from the TGF-β1:TβRII:TβRI ternary complex reported by Radaev (PDB 3KFD) [3]; to simplify the presentation, the bound TβRI is not displayed.

Rat Serum Albumin
Signal Peptide

Rat BG_E G24 ...

MKWVTFLLLL FISGSAFSAA AGPEPSTRCE LSPINASHPV QALMESFTVL SGCASHGTTG 60

LPREVHVLNL RSTDQGPGQR QREVTLHLNP IASVHTHHKP IVFLLNSPQP LVWRLKTERL 120

AAGVPRLFLV SEGSVVQFPS GNFSLTAETE ERNFPQENEH LLRWAQKEYG AVTSFTELKI 180

ARNIYIKVGE DQVFPPTCNI GKNFLSLNYL AEYLQPKAAE GCVLPSQPHE KEVHIIELIT 240

PSSNPYSAFQ VDIIVDIRPA QEDPEVVKNL VLILKSKKS VNWVIKSFDV KGNLKVIAPNS 300

IGFGKESER SMTMTKLVRD DIPSTQENLM KWALDAGYRP VTSYTMAPVA NRFHLRLENNE 360

Rat BG_E ...D383 Human TβRII P48 ...

EMRDEEVHT IPPELRILLD PDKLPQLCKF CDVRFSTCDN QKSCMSNCSI TSICEKPQEVC 420

VAVWRKNDE NITLETVCHD PKLPYHDFIL EDAASPKCIM KEKKKPGETF FMCSCSSDECN 480

Human TβRII ...D159

DNIIFSEEY NTSNPDHHHH HH 502

Region with no consistent pattern of predicted secondary structure, prior to the first predicted β-strand in the betaglycan endoglin domain that begins with R30 and ends with S34 (Mendoza, et. al, Biochemistry, 48, 11755-11765, 2003).

Region with no consistent pattern of predicted secondary structure, past the last predicted β-strand in the endoglin domain, which begins with R353 and ends with L358 (Mendoza, et. al, Biochemistry, 48, 11755-11765, 2009).

Artificial linker that connects the betaglycan endoglin and TβRII domains

Region with NMR-derived Lipari-Szabo backbone order parameters less than 0.6 (Deep, et. al, Biochemistry 42, 10126-10139, 2003). Lipari-Szabo backbone order parametersprovide information about backbone flexibility on the ns-ps timescale, with 1.0 being totally rigid on this timescale, and 0.0 being totally flexible.

Supplementary Figure S2: Betaglycan endoglin domain-TβRII (ER) trap as produced in a CMV-based vector. Potential N-linked glycosylation sites are indicated by an asterisk below the corresponding arginine (N) residue.

```
Rat Serum Albumin
  Signal Peptide
                                   Human TβRII N42 ...
MKWVTFLLLL FISGSAFSAA ANGAVKFPQL CKFCDVRFST CDNQKSCMSN CSITSICEKP   60

QEVCVAVWRK NDENITLETV CHDPKLPYHD FILEDAASPK CIMKEKKKPG ETFFMCSCSS  120

     Human TβRII ...D159                           Rat BGE G24 ...
DECNDNIIFS EEYNTSNPDG LGPVESSPGH GLDTAAAGPE PSTRCELSPI NASHPVQALM  180

ESFTVLSGCA SHGTTGLPRE VHVLNLRSTD QGPGQRQREV TLHLNPIASV HTHHKPIVFL  240

LNSPQPLVWR LKTERLAAGV PRLFLVSEGS VVQFPSGNFS LTAETEERNF PQENEHLLRW  300

AQKEYGAVTS FTELKIARNI YIKVGEDQVF PPTCNIGKNF LSLNYLAEYL QPKAAEGCVL  360

PSQPHEKEVH IIELITPSSN PYSAFQVDII VDIRPAQEDP EVVKNLVLIL KSKKSVNWVI  420

KSFDVKGNLK VIAPNSIGFG KESERSMTMT KLVRDDIPST Q[illegible]LMKWALD AGYRPVTSYT  480

                            Rat BGE ...D383     Human TβRII P48 ...
MAPVANRFHL RLENNEEMRD EEVHTIPPEL RILLDPDKLP QLCKFCDVRF STCDNQKSCM  540

SNCSITSICE KPQEVCVAVW RKNDENITLE TVCHDPKLPY HDFILEDAAS PKCIMKEKKK  600

                   Human TβRII ...D159
PGETFFMCSC SSDECNDNII FSEEYNTSNP DHHHHHH                            637
```

Artificial linker that connects TβRII and the betaglycan endoglin domain

Region with no consistent pattern of predicted secondary structure, prior to the first predicted β-strand in the betaglycan endoglin domain, that begins with R30 and ends with S34 (Mendoza, et. al, Biochemistry, 48, 11755-11765, 2003).

Region with no consistent pattern of predicted secondary structure, past the last predicted β-strand in the endoglin domain, which begins with R353 and ends with L358 (Mendoza, et. al, Biochemistry, 48, 11755-11765, 2009).

Artificial linker that connects the betaglycan endoglin and TβRII domains

Region with NMR-derived Lipari-Szabo backbone order parameters less than 0.6 (Deep, et. al, Biochemistry 42, 10126-10139, 2003). Lipari-Szabo backbone order parametersprovide information about backbone flexibility on the ns-ps timescale, with 1.0 being totally rigid on this timescale, and 0.0 being totally flexible.

Supplementary Figure S3: TβRII-Betaglycan endoglin domain-TβRII (RER) trap as produced in a CMV-based vector. Potential N-linked glycosylation sites are indicated by an asterisk below the corresponding arginine (N) residue.

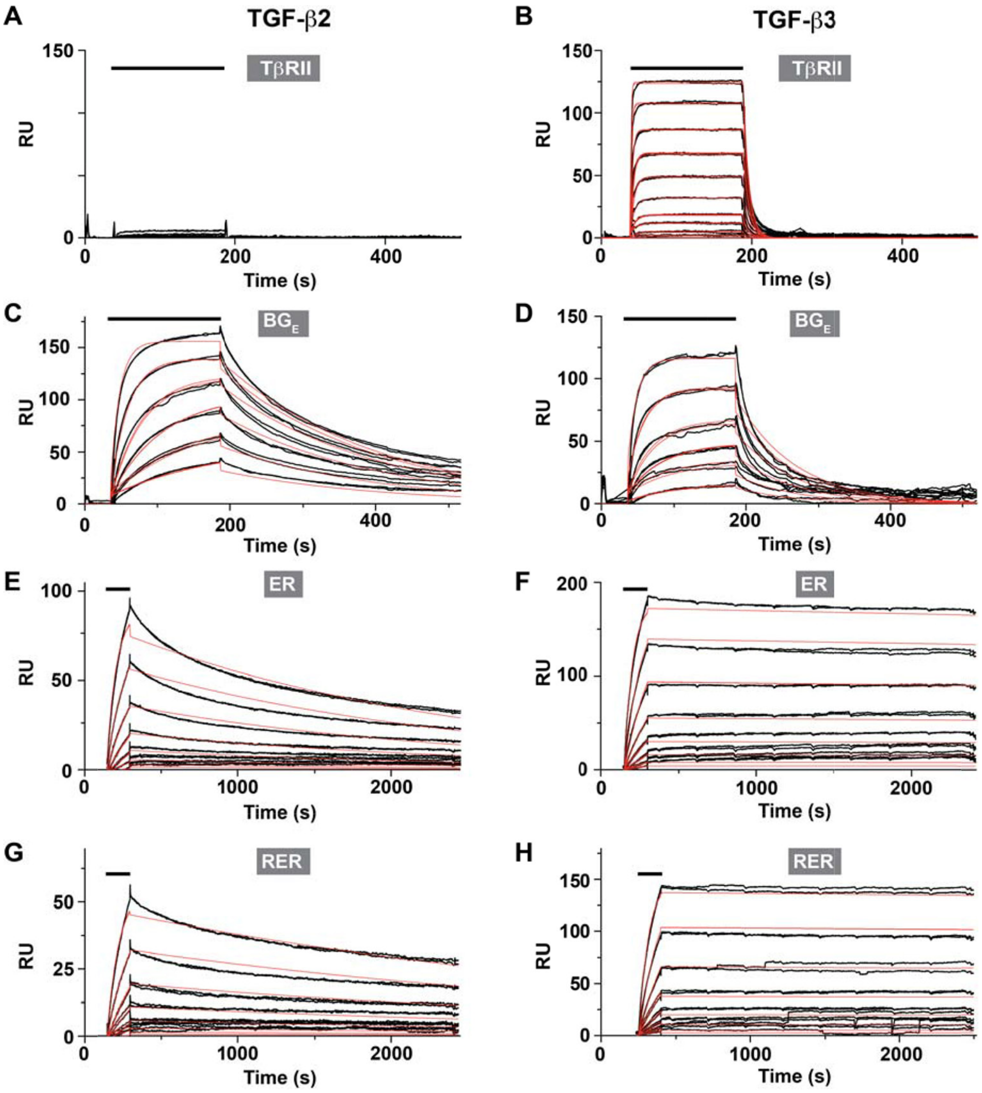

Supplementary Figure S4: Binding of receptor traps, and component domains, to TGF-β2 and TGF-β3. (**A**, **B**) Injection of a two-fold dilution series of TβRII (0.125–4 μM) over immobilized TGF-β2 (A) and TGF-β3 (B). Injections were performed in triplicate. Raw sensorgrams are shown in black. Global fit of the raw data to a 1:1 binding model is shown as smooth red curves (**C**, **D**) Injection of the betaglycan endoglin domain, BG_E, as a two-fold dilution series (0.125–4 μM) over immobilized TGF-β2 and TGF-β3. Other details are as in panels A and B. (**E**, **F**) Injection of the ER receptor trap as a two-fold dilution series (12.5–400 pM) over immobilized TGF-β2 and TGF-β3. Other details are as in panels A and B. (**G**, **H**) Injection of the RER receptor trap as a two-fold dilution series (12.5–400 pM) over immobilized TGF-β2 and TGF-β3. Other details are as in panels A and B.

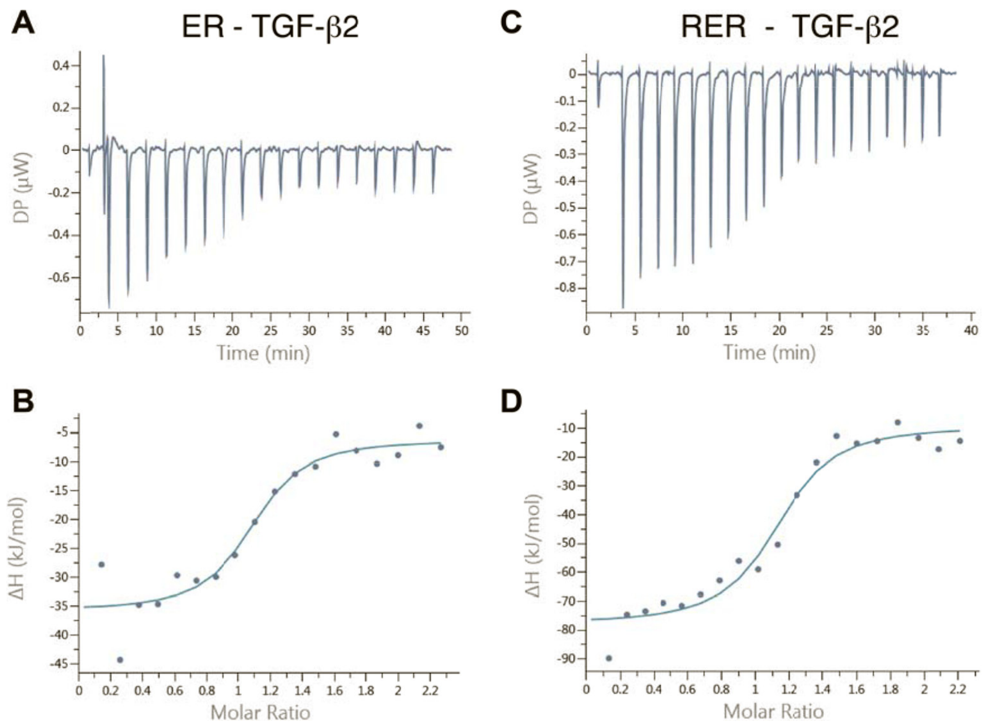

Supplementary Figure S5: Receptor trap binding stoichiometry and ability to block receptor binding. (**A**, **C**). ITC traces of the raw heat values for titration of 100 μM ER into 8.5 μM TGF-β2 (A) or 54 μM RER into 5 μM TGF-β2 (C). Titrations were performed at 37°C at pH 7.0 in the presence of 30 mM CHAPS. (**B**, **D**). Integrated heat values (data points) fitted to a standard binding isotherm for 1:1 binding (smooth curve) for ER or RER binding to TGF-β2 (B and D, respectively).

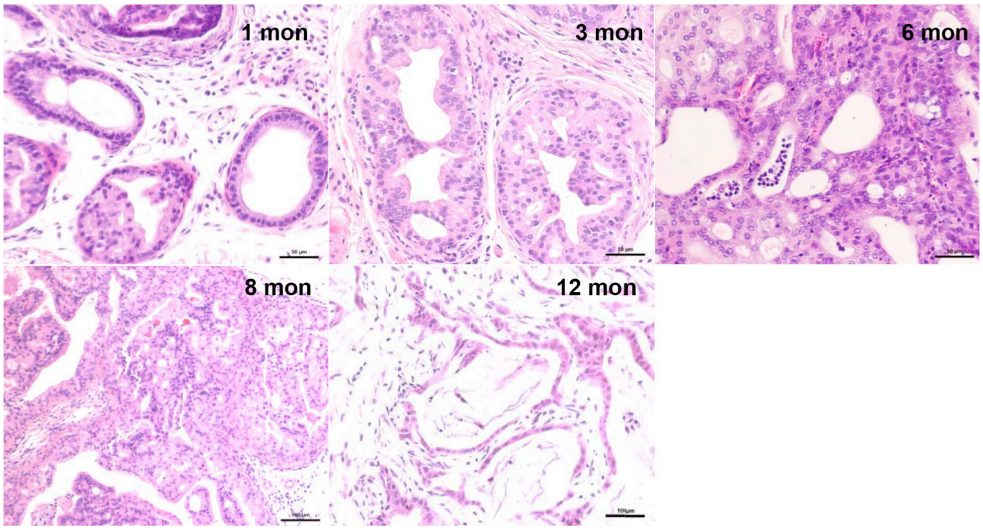

Supplementary Figure S6: H&E staining of DP prostate glands of PTEN KO mice in different age stages as indicated. Scale bar represents 100 μm.

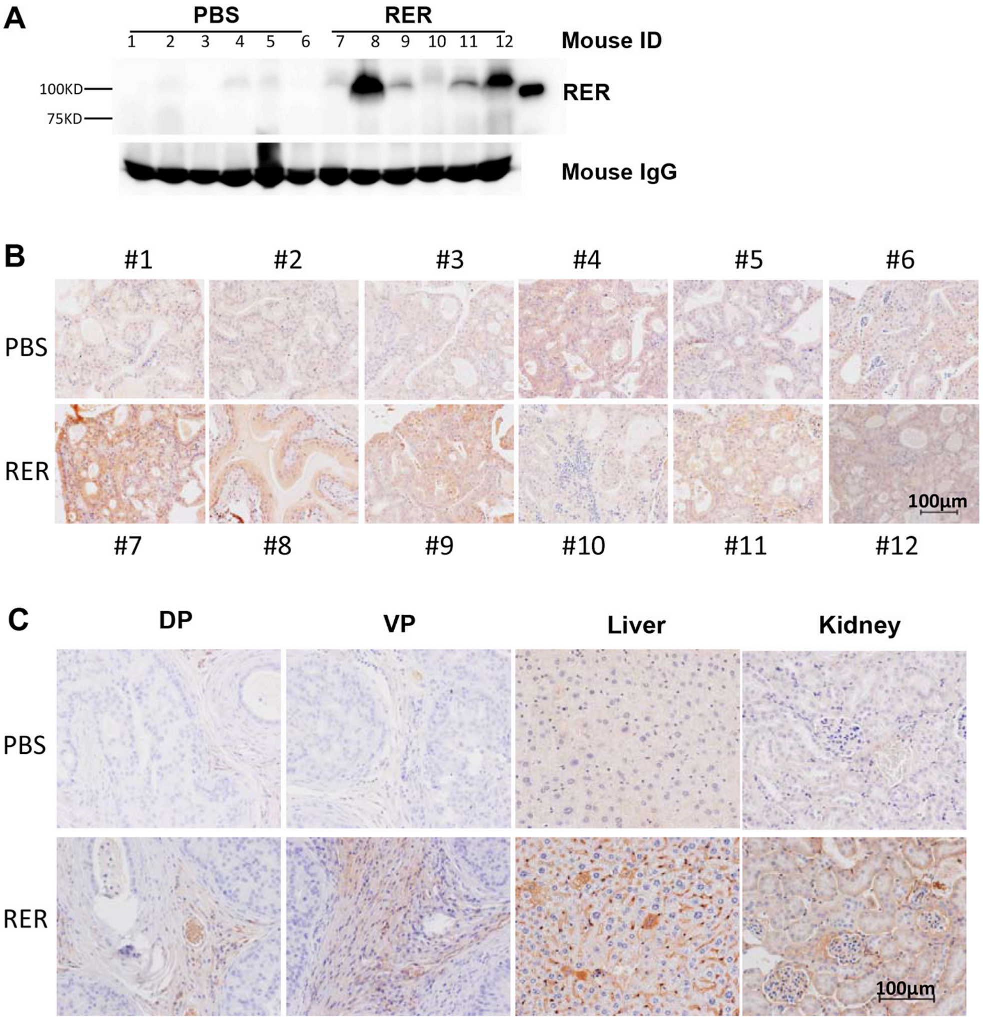

Supplementary Figure S7: RER was distributed over multiple organs after i.p. injection in the Pten conditional knockout mice. (**A**) Western blot analysis of RER detection by TGF-β type II receptor antibody in the mouse serum. Mouse IgG heavy chain protein level was used to validate equal sample loading. (**B**) IHC analysis of RER in anterior prostate (AP). (**C**) IHC analysis of RER in ventral prostate (VP), dorsolateral prostate (DLP), liver and kidney tissue of the PBS or RER injected mice.

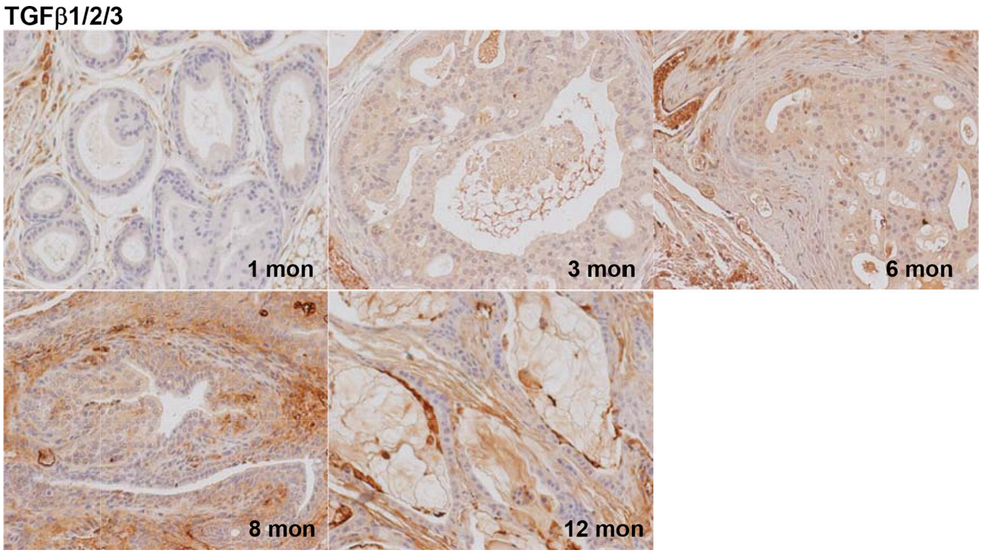

Supplementary Figure S8: IHC staining of TGFβ1/2/3 in DP glands of PTEN KO mice in different age stages as indicated. The brown color density represents the levels of TGFβ expression.
